# Disaggregated level child morbidity in Bangladesh: An application of small area estimation method

**DOI:** 10.1371/journal.pone.0220164

**Published:** 2020-05-20

**Authors:** Sumonkanti Das, Bappi Kumar, Luthful Alahi Kawsar

**Affiliations:** 1 Department of Quantitative Economics, Maastricht University, Maastricht, The Netherlands; 2 Department of Statistics, Shahjalal University of Science & Technology, Sylhet, Bangladesh; 3 Faculty of Engineering and Information Sciences, University of Wollongong, Wollongong, NSW, Australia; Addis Ababa University School of Public Health, ETHIOPIA

## Abstract

Acute respiratory infection (ARI) and diarrhoea are two major causes of child morbidity and mortality in Bangladesh. National and regional level prevalence of ARI and diarrhoea are calculated from nationwide surveys; however, prevalence at micro-level administrative units (say, district and sub-district) is not possible due to lack of sufficient data at those levels. In such a case, small area estimation (SAE) methods can be applied by combining survey data with census data. Using an SAE method for the dichotomous response variable, this study aims to estimate the proportions of under-5 children experienced with ARI and diarrhoea separately as well as either ARI or diarrhoea within a period of two-week preceding the survey. The ARI and diarrhoea data extracted from Bangladesh Demographic and Health Survey 2011 are used to develop a random effect logistic model for each of the indicators, and then the prevalence is estimated adapting the World Bank SAE approach for the dichotomous response variable using a 5% sample of the Census 2011. The estimated prevalence of each indicator significantly varied by district and sub-district (1.4–11.3% for diarrhoea, 2.2–11.8% for ARI and 4.3–16.5% for ARI/diarrhoea at sub-district level). In many sub-districts, the proportions are found double of the national level. District and sub-district levels spatial distributions of the indicators might help the policymakers to identify the vulnerable disaggregated and remote hotspots. Particularly, aid industries can provide effective interventions at the highly vulnerable spots to overcome the gaps between micro and macro level administrative units.

## Introduction

Diarrhoea and acute respiratory infection (ARI) are recognized as important causes of global child morbidity and mortality. Pneumonia (a form of ARI) and diarrhoea remain major causes of child death. The impact of these two diseases is about 29% of all under-5 child deaths causes loss of 2 million young lives each year [1]. Pneumonia alone accounts for about 16% of these young child deaths. The number of deaths due to pneumonia can be reduced by the early diagnosis and treatment of ARI. The integrated Global Action Plan for Pneumonia and Diarrhoea (GAPPD) aims to reduce mortality from pneumonia and diarrhoea among under-5 children to fewer than 3 per 1000 and 1 per 1000 live births respectively by 2025 [2]. The United Nation (UN) has set a target under the third sustainable development goal (SDG) to end the epidemics of water-borne diseases and other communicable diseases by 2030 (target 3.3) to achieve the SDG target of ending preventable deaths of under-5 children to reduce child mortality to below 25 per 1,000 live births (target 3.2). Recently WHO reported that under-5 child mortality rates reduced to 41 per 1000 live births in 2016 from 93 per 1000 live births in 1990, however still now every day on an average 15,000 children die before reaching their fifth birthday [3]. About half of these under-5 child deaths can be prevented through simple and cost-effective interventions at proper time.

As per GAPPD, the solutions to tackle pneumonia and diarrhoea do not require major advances in technology since existing interventions have already proven its effectiveness. Children are dying because the provided services are not enough to meet the demand and children at higher risk are not being reached. Use of effective interventions such as exclusive breastfeeding for the first 6 months, proper life-saving treatment for the children with suspected pneumonia and providing oral rehydration therapy to the children with diarrhoea are sufficient to achieve these goals [2]. Thus, identifying the children at greatest risk, hardest to reach and most neglected, and targeting them with interventions of proven efficacy will enable us to close the gap, and ultimately to end the heavy toll of preventable child deaths.

The prevalence of ARI and diarrhoea at national and divisional levels are usually estimated from a nationwide household survey. In Bangladesh, such nationwide data on ARI and diarrhoea information are collected through household surveys conducted by the Bangladesh Bureau of Statistics (BBS), and through the Demographic and Health Survey (DHS). Diarrhoea and ARI data are collected by asking mothers whether their children experienced a diarrhoea episode or ARI symptom during two weeks preceding the survey. According to the seven Bangladesh DHS (BDHS) surveys from 1993 to 2014, the prevalence rates of diarrhoea and ARI have been improved in Bangladesh throughout 1990–2010, however, there was no steady declining trend. The prevalence of diarrhoea was around 20% for the period of 1993–2004 with a decline only in 1996 (about 13%); however, the rate was found stable at around 5% in the later surveys. For ARI, there was a somewhat steady decline in the prevalence over the period except in 2007, when the rate was unexpectedly jumped to about 10% [4]. In the last 2014 BDHS, the episodes of ARI and diarrhoea were found around 5% for both cases [4], while the rates were 6% and 5% respectively in 2011 BDHS [5]. The distribution of diarrhoea and ARI prevalence at division level also does not show any specific declining trends [4–10]. Collection of survey data at different time-periods may be one of the reasons, which suggest some seasonal effects in the prevalence. An approximate declining trend in both diarrhoea and ARI prevalence are observed only for *Rajshahi* (and also *Rangpur*) division during 1993–2014 period. Interestingly, water-prone divisions *Chittagong*, *Barisal* and *Sylhet* were most likely vulnerable to diarrhoea and ARI diseases in most of the surveys. Although division level estimates of diarrhoea and ARI prevalence are estimable from the survey data, those at disaggregated levels (such as district and sub-district) are not estimable solely from the survey data due to the limited number of observations at the desired micro level. Consequently, the government cannot find the disaggregated hotspots highly vulnerable to ARI and diarrhoea. Identifying such hotspots might help the aid industries concerned targeting efficient interventions.

A few district-level studies on diarrhoea prevalence are found in the literature. In a cross-sectional study covering seven vulnerable districts of Bangladesh which are prone to cyclone, flood, and salinity (*Bagerhat*, *Barguna*, *Cox’s Bazar*, *Faridpur*, *Khulna*, *Satkhira*, and *Sirajganj*), 10.3% under-5 children (95% CI 9.16–11.66) experienced with diarrhoea in 2012 during the preceding month of interview [11]. Though they have district-specific data, they did not report district-specific estimates of diarrhoea prevalence. No study has also been found conducted at the sub-district or lower administrative units. Most of the studies conducted by the International Centre for Diarrhoeal Disease Research, Bangladesh (ICDDR’B) are related to environmental and clinical risk factors of different types of diarrhoeal diseases [12, 13]. Using BDHS data, there are studies on modelling diarrhoea or ARI prevalence based on the logistic or logistic mixed model for determining their risk factors [14, 15], not for prediction purpose since information of the risk factors are not available in the census data.

Since districts and sub-districts are ignored in the sampling design for the nationally representative household survey, the estimates of diarrhoea and ARI prevalence or any other target parameters are not estimable at these levels. The BDHS 2011 data covers all the 64 districts but only 396 out of 544 sub-districts. Since the sample sizes both at district and sub-district levels are too small to have efficient estimates, the design-based direct estimates are not reasonable to use [16]. Consequently, the policymakers are unable to do their plan focusing on the district or its lower administrative hierarchies. Small area estimation (SAE) is a statistical technique to obtain estimates of a target parameter with better precision for disaggregated administrative units of a country. The basic idea of the SAE method is to model survey data statistically [16]. Such modelling may include use of a recent census or administrative data. Survey data consist of the target variable and a regression model is specified with some explanatory variables which are common in both survey and census data.

The SAE methods are broadly two types based on the availability of the explanatory information. If unit-level explanatory variables (such as children level variables used in this study) are available for all the population units (or a sample of the census), SAE methods use unit-level models such as nested error regression model [17]. While if area-level aggregate statistics extracted from census or any administrative data source are available, area-level SAE methods apply area-level models such as Fay-Herriot model [18]. One of the major problems of area-level methods is that the estimated standard errors are assumed known, though there are always a significant number of small areas (say here sub-districts) with unreliable standard errors since they are calculated based on small sample sizes. Also for some small areas, the estimates with their standard errors are not available due to zero observations. In addition, area-level model-based SAE method can be used for estimating the prevalence only at that level or its higher-level (such as division level). However, it is not possible to estimate prevalence at lower administrative units like sub-district. The unit-level SAE method has advantages for avoiding the two abovementioned problems and so a unit-level SAE approach has been followed to estimate the prevalence of the considered health indicators in this study.

The World Bank has been utilising a unit-level SAE method known as ELL after the authors Elbers, Lanjouw, and Lanjouw [19] for poverty and nutrition mapping in many developing countries including Bangladesh [20, 21]. The basic idea of the ELL methodology is to develop a regression model using a continuous response variable (such as per capita consumption expenditure, weight-for-age Z-score) from survey data and apply it to a census or administrative data source. Since the variable of interest for diarrhoea or ARI prevalence is dichotomous (whether a child has experienced with diarrhoea or not during a period) instead of continuous, the ELL methodology cannot be implemented without modification. However, the basic idea can be implemented after developing a generalized linear mixed model (GLMM), more specifically, a random effect logistic model for the dichotomous response variable [22, 23, 24, 25]. The SAE methods based on GLMM model have been applied to estimate district-level institutional births [23] and unmet need for contraception [24] in Ghana, and disaggregated (district and sub-district) level diarrhoea prevalence in Nepal [25]. The main difficulty is to develop a proper GLMM model incorporating the available survey data with a recent census or administrative data for a country. Another problem arises in developing a proper logistic model when the outcome is less frequent among the study population [26]. In this study, developing a proper (mixed effect) logistic model with sufficient explanatory variables was difficult since the occurrence of diarrhoea (also for ARI) was found only for about 5% children in the BDHS 2011 data.

Recently Das, Chandra and Saha [27] conducted an SAE study on district-level diarrhoea prevalence in Bangladesh employing an area-level SAE method. District-specific direct estimates of diarrhoea prevalence with their standard errors were calculated based on the design-based direct estimator using the data of BDHS 2014. These direct estimates were used as the response variable and some district-specific variables collected from the Census 2011 reports were used as the explanatory variables. Though division-level diarrhoea prevalence can be calculated from these district-level estimates, it is not possible to estimate sub-district-specific diarrhoea prevalence. For estimating sub-district level diarrhoea prevalence, sub-district-specific (or lower administrative level) Fay-Herriot model is required to develop that might be difficult due to lack of efficient direct estimates and their standard errors (which are assumed known in the area-level model) at that level. Thus, the main aim of this study is to estimate the prevalence of ARI and diarrhoea for under-5 children at district and sub-district levels using a unit-level SAE technique for the dichotomous response variable. In addition, the aims include estimating the proportion of children suffering from at least one of the two indicators during the 2-week period (hereafter refereed as ARI/diarrhoea) at disaggregated levels. Finally, disaggregated level spatial distributions of all the three indicators are mapped to highlight the most vulnerable hotspots. The rest of the paper is set up as follows. Section 2 describes survey and census data used in this study; Section 3 describes a design-based direct estimator and a model-based SAE estimator for binary response variable based on the ELL method; Section 4 illustrates the fitted models, spatial distribution of the prevalence, and explores the characteristics of the considered SAE estimator; and concluding remarks are given in Section 5.

## Data description

Diarrhoea and ARI data used in this study are extracted from the BDHS 2011 survey. The main reasons for using BDHS 2011 (instead of recent BDHS 2014) is its concurrency with the Census 2011 and also the hierarchy of the administrative units are available down to sub-district (sub-districts are not available in the BDHS 2014). The data of BDHS 2011 are collected following a two-stage stratified sampling design by covering all 7 divisions, 64 districts, and 396 (out of 544) sub-districts. In the BDHS 2011, ARI and diarrhoea information are available for a total of 8341 children [5].

Full census data of Bangladesh is unavailable for academic purposes; however, a 5% sample of the full census data is available from the BBS. Socio-demographic characteristics such as age, sex, education, schooling, employment, disability and housing characteristics such as house type, source of drinking water, and household sanitation are available in the census data. A number of contextual variables at district and sub-district levels are created using the individual and household level data of the 5% census data. In the model specification, these contextual variables are used in addition to those variables at children level common in both survey and census data. The contextual variables used in the model development for capturing the variation at the district and sub-district levels are shown in S1 and S2 Tables. In model development, two-way interactions of residence, division, sex, and age are utilized to develop the best models. The interaction of division and residence also covers the sampling design of the BDSH.

The structure of administrative units and the distribution of children in BDHS 2011 and Census 2011 are shown in Table 1. The mean and minimum number of children at district (137 and 15) and sub-district (22 and 1) levels indicate that reliable estimates are not possible from the BDHS survey at these disaggregated levels, particularly at the sub-district level. Since the BDHS data is collected through cluster sampling design and cluster-specific multilevel models are considered to be developed, cluster-specific information are also examined. Table 1 shows that the mean number of children at the cluster level in the survey is higher than that in the census data. As per the definition of enumeration area (EA) which is the cluster in the survey, an EA/cluster consists of on average 110–120 households. In the survey, 30 households were covered from each of the sampled clusters, while 5% of the 110–120 households are selected in the 5% census data. As a consequence, the cluster-specific mean number of under-5 children is very low in the considered census data.

**Table 1:** Structure of administrative units and children in the Census 2011 and BDHS 2011.

	5% of 2011 Census				2011 BDHS			
	Division	Zila	Upzila	EA	Division	Zila	Upzila	EA
**Administrative Units**	7	64	544	291669	7	64	396	600
**Mean Children U5**	107415	11749	1382	3	1250	137	22	15
**Minimum Children U5**	43026	2504	41	1	977	15	1	1

The number of children experienced with diarrhoea is found zero for about three-fifths clusters (351 out of 600), for ARI and ARI/diarrhoea the proportions are respectively 50.1% and 32.3% (301 and 194 out of 600). These practical issues would be problematic for developing an appropriate multilevel logistic model. This issue is discussed in the model development section.

## Statistical methodology

Let the occurrence of an outcome for *k*^*th*^ child belonging to *j*^*th*^ cluster of *i*^*th*^ area is denoted by *y*_*ijk*_, which takes value 1 if the child experienced the outcome (say diarrhoea) preceding the last two weeks of the survey date and 0 if the child did not experience the outcome. The target is to calculate division, district and sub-district level proportions of under-5 children who experienced a target outcome during the period. Since the survey data is representative at the division level, design-based direct estimator might provide unbiased and consistent estimates at the division level but not at the other two disaggregate levels. Therefore, an appropriate SAE estimator has been applied for estimating proportions at all three levels. The design-based direct estimator and a unit-level SAE estimator for binary response variable are briefly explained in the following two sub-sections.

### Design-based direct estimator

The Horvitz-Thompson estimator for estimating mean and total of a super-population in a stratified sample is utilized to obtain design-based direct estimates of target parameter for small areas using the response values available in the survey data. The design-based direct estimator (denoted by DIR) for the target proportion *P*_*i*_ for the area *i* is

$${\hat{P}}_{i}^{DIR}=\sum_{k\in s_{i}}w_{ik}y_{ik},i=1,\ldots ,D$$

where $w_{ik}=w_{ik}^{*}/\sum_{k\in s_{i}}w_{ik}^{*}$ is normalized survey weights for *k*^*th*^ child belonging *i*^*th*^ district with $\sum_{k\in s_{i}}w_{ik}=1$ and $w_{ik}^{*}$ is the survey weight (inverse of the inclusion probability). Following Särndal *et al*. [28], the design-based variance of the direct estimator ${\hat{P}}_{i}^{Dir}$ can be approximated by,

$$var({\hat{P}}_{i}^{DIR})\approx {\sum_{k\in s_{i}}w_{ik}(w_{ik}-1)(y_{ik}-{\hat{P}}_{i}^{DIR})}^{2}.$$

This direct estimator is design-unbiased but based on the non-representative area-specific sample data. Consequently, the direct estimator becomes unreliable due to area-specific small sample size and also for some areas with no sample data. Though all the districts are covered in BDHS 2011 data, sample sizes for a significant number of districts are very small since only a few clusters are covered for those districts. Also for some districts, the number of children experienced with an outcome (say, diarrhoea) is found zero, which provides zero prevalence and zero standard error. The model-based SAE methods that ‘borrow strength’ via statistical models overcome these practical issues for calculating reliable small area estimates [29].

### Model-based SAE estimator

Suppose *p*_*ijk*_ = *P*(*y*_*ijk*_ = 1) represents the probability of having diarrhoea for *k*^*th*^ child belonging to *j*^*th*^ cluster of *i*^*th*^ area. The first target is to develop a nested-error logistic regression model which is a special case of the generalized linear mixed model (GLMM) as

$$\mathrm{logit}\left(p_{ijk}|x_{ijk},u_{ij}\right)=\log\left[p_{ijk}/\left(1-p_{ijk}\right)\right]=x_{ijk}^{T}\beta +u_{ij}$$

where **x**_*ijk*_ is vector of explanatory values, **β** is vector of regression parameters, and *u*_*ij*_ corresponds to cluster-specific random errors respectively. The random errors are usually assumed to be independent and identically distributed with mean zero and constant variance $\left(\sigma_{u}^{2}\right)$. The regression parameters **β** and variance component $\sigma_{u}^{2}$ can be estimated via the restricted maximum likelihood method. The regression model can be extended to a higher level (such as area-specific effect), however the ELL methodology assumes heterogeneity at cluster-level rather than target area-level [19]. For estimating the target parameters with their root mean squared error (RMSE), the estimated regression coefficients, variance components, residuals, and the explanatory information for all the under-5 census children are used as input in a parametric or non-parametric bootstrap procedure of the ELL method. The basic steps of the parametric or non-parametric bootstrap procedure are briefly explained following Das and Haslett [30].

Step 1: fit random effect logistic model to obtain regression coefficients $\hat{\beta }$ with their estimated variance-covariance matrix $\hat{v}\left({\hat{\beta }}_{gls}\right)$, and cluster-specific residuals with the variance component ${\hat{\sigma }}_{u}^{2}$.
Step 2: generate regression parameters β* from a suitable sampling distribution say the multivariate normal distribution $N\left({\hat{\beta }}_{gls},\hat{v}\left({\hat{\beta }}_{gls}\right)\right)$;
Step 3: generate cluster-specific random errors (except the children level) from a suitable parametric distribution such as $N\left(0,{\hat{\sigma }}_{u}^{2}\right)$ or t-distribution. In case of a non-parametric bootstrap procedure, draw cluster-specific random errors by resampling via simple random sampling with replacement (SRSWR) from their empirical distribution (i.e., from the estimated cluster-specific sample residuals stored in Step 1);
Step 4: generate bootstrap response values $p_{ijk}^{*}$ using the generated regression parameters and the cluster-specific random errors. The generated response values $p_{ijk}^{*}$ are then aggregated to estimate the area-specific parameter of interest, say, $P_{i}^{*}=N_{i}^{-1}\sum_{j=1}^{C_{i}}\sum_{k=1}^{N_{ij}}p_{ijk}^{*}$ where *N*_*i*_ and *N*_*ij*_ are area and cluster-specific number of under-5 census children.

The steps 2–4 are iterated for a large number of times say *B* = 500 and then the mean and the standard deviation of these *B* estimates are considered as the final estimates and their RMSEs respectively as

$${\hat{P}}_{i}^{ELL}=B^{-1}\sum_{b=1}^{B}P_{i}^{*}\;\mathrm{and}\;rmse_{i}^{ELL}=\sqrt{B^{-1}\sum_{b=1}^{B}{\left(P_{i}^{*}-{\hat{P}}_{i}^{ELL}\right)}^{2}}.$$

This estimator is denoted by ELL in the text since the estimator is developed based on the ELL approach but used for a binary response variable. In the original development of ELL method for a continuous response variable, heteroscedasticity has been considered at the unit-level (say, children). If the response variable is weight-for-age Z-score, this heteroscedasticity issue can be employed. Since the response variable is binary, the heteroscedasticity at the unit-level is not considered here.

## Results

Fixed-effect logistic models (referred to as GLM) and random intercept logistic models (referred to as GLMM) are developed using children demographic characteristics, household characteristics, place of residence, regional settings and some contextual variables. The final selected models for these three health indicators are shown in S1 Table. The final models with their corresponding inputs are utilized in the ELL approach to estimate the proportions of each health indictor with their RMSEs and coefficients of variation (CVs). Table 2 shows that cluster-specific two-level GLMM models perform better than the fixed-effect GLM model in terms of AIC, Likelihood Ratio Test (LRT), and area under the ROC curve (referred to as AUC) for each of the three child health indicators. The AUC values indicate that the GLMM can classify children health status more correctly than the GLM in all cases; particularly for ARI, about 73% of children are correctly classified.

**Table 2:** Summary statistics and diagnostics of the fitted logistic (GLM) and random intercept logistic (GLMM) models for diarrhoea, ARI, and ARI/diarrhoea prevalence, BDHS 2011 ♀.

Indicators	n	P	Model	AIC	logLik	$\sigma_{u}^{2}$	LRT of $H_{0}:\sigma_{u}^{2}=0$	AUC (%)
**Diarrhoea**	8341	21	GLM	3111.8	-1534.9	-	*χ*^2^: 3.7120	54.86
			GLMM	3110.1	-1533.0	0.1687	p-value: 0.0215	71.55
**ARI**	8341	22	GLM	3680.2	-1818.1	-	*χ*^2^: 6.2343	61.11
			GLMM	3676.0	-1815.0	0.1921	p-value: 0.0063	72.26
**ARI/Diarrhoea**	8341	21	GLM	5371.6	-2664.8	-	*χ*^2^: 2.7519	58.41
			GLMM	5370.8	-2663.4	0.0791	p-value: 0.0448	65.78

**♀** n = sample size, p = # of covariates, $\sigma_{u}^{2}$ = cluster-level variance component, LRT = likelihood ratio test, AUC = Area under the receiving operating characteristics curve.

Although the cluster-specific random errors are assumed to follow the normal distribution, the normality assumption is not satisfied for diarrhoea and ARI separately, however, the normality assumption is approximately satisfied for ARI/diarrhoea (see Q-Q plots in S1 Fig). The distribution of clusters with zero prevalence (58.1% and 50.2% respectively for diarrhoea and ARI) may be one of the reasons for such non-normal distribution of residuals obtained from the models of diarrhoea and ARI prevalence. Although the residual distributions are found non-normal, their variances are found approximately homogeneous (see distributions of residuals in S1 Fig). To avoid the impact of non-normal residuals, non-parametric bootstrap procedure assuming constant variance of residuals are employed in the prediction of these health indicators.

**Fig 1:**
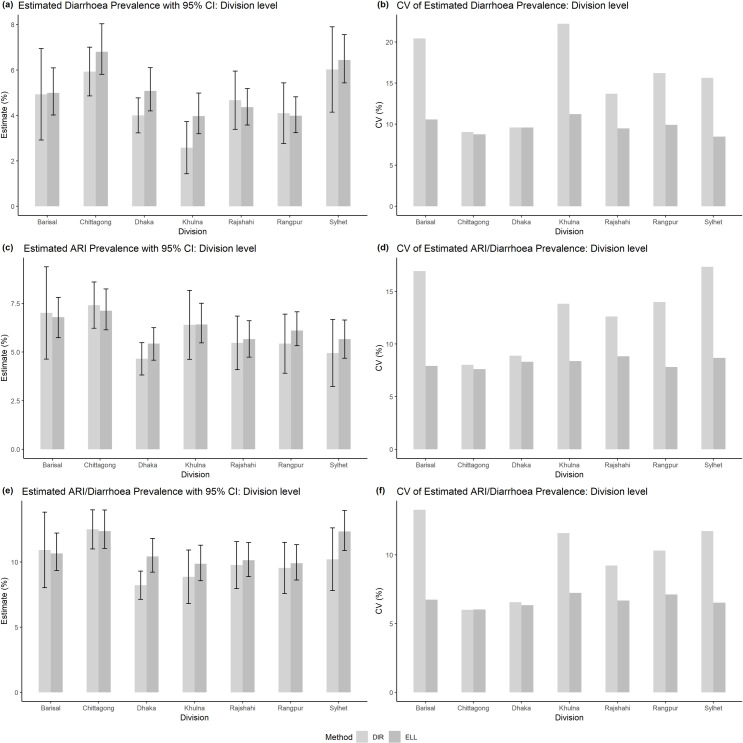
Prevalence of diarrhoea, ARI, and ARI/diarrhoea (plots (a), (c), (e) respectively) among under-5 children at division level in Bangladesh with their 95% CI and also their coefficient of variations (CV) (plots (b), (d), (f) respectively) estimated by the direct (light grey bars) and ELL (grey bars) estimators.

To examine the performance of the SAE estimator at the higher administrative levels, division level estimates are estimated and compared with the estimates calculated by the design-based DIR estimator. The bar plots with 95% confidence interval (CI) shown in Fig 1 (plots (a), (c) and (e)) indicate that the ELL estimator provides very similar estimates as the DIR estimator at division level, however the ELL estimator shows higher accuracy measured by CV (in %). The bar plots of CVs by division in Fig 1 (plots (b), (d) and (f)) show that the ELL estimator provides considerably lower CVs than the DIR estimator for most of the divisions except *Dhaka* and *Chittagong* division, where the number of sampled children is considerably higher than the other divisions. The 95% CI lines also show that the DIR estimator provides higher confidence interval for most of the divisions due to higher standard errors.

The division level prevalence of diarrhoea and ARI estimated by the ELL estimator indicate that the children who live in *Chittagong* division are highly vulnerable to both indicators (about 6.5% and 7.5%respectively), followed by those who live in *Sylhet* and *Barisal* divisions are vulnerable to diarrhoea (6.4%) and ARI (6.8%) respectively. The bar plot of ARI/diarrhoea indicator shows that children of these three divisions (12.4% for Chittagong, 12.3% for Sylhet, and 10.7% for Barisal) are highly vulnerable compared to those living in other divisions. The children of *Khulna* division have a lower prevalence of diarrhoea (about 4.0%), however they are more affected by ARI (about 6.4%). On the other hand, children of Dhaka division are equally vulnerable to the occurrence of diarrhoea (5.1%) and ARI (5.4%). Consequently, the bar plot corresponds to ARI/diarrhoea indicates that about 10% children living in *Dhaka* and *Khulna* divisions have experience with either ARI or diarrhoea. The prevalence of all three indicators are very similar for the children of *Rajshahi* and *Rangpur* divisions.

**Fig 2:**
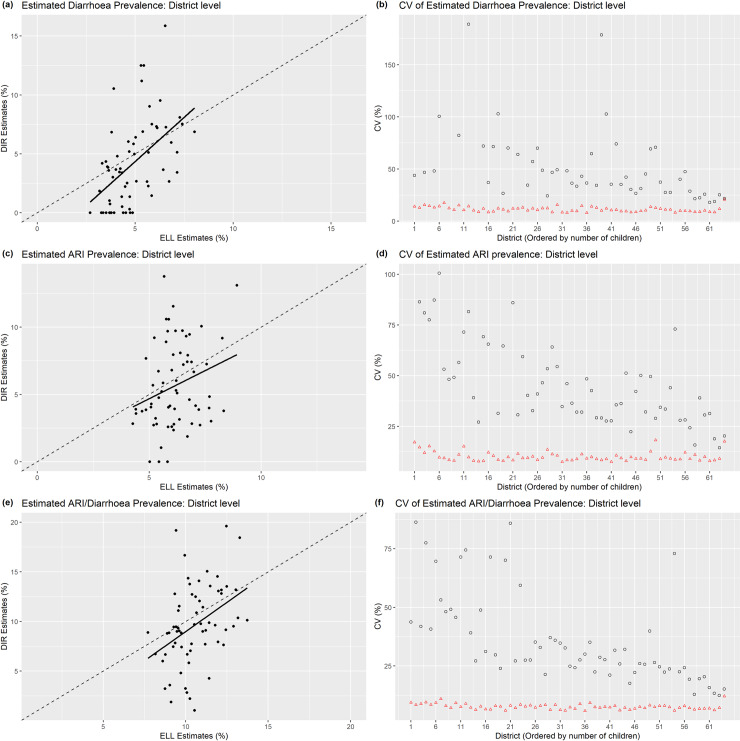
Bias diagnostic plot with y = x line (dashed) and regression line (solid) for estimated prevalence of diarrhoea, ARI, and ARI/diarrhoea among under-5 children at district level in Bangladesh (plots (a), (c), (e) respectively) by the ELL estimator and the corresponding coefficient of variations (CV) against the district index ordered by the number of total children (plots (b), (d), (f) respectively) estimated by the direct (black circle) and ELL (red triangle) estimators.

The district-level prevalence of diarrhoea, ARI and ARI/diarrhoea estimated by the ELL estimator are plotted against those prevalence estimated by the DIR estimator for assessing the unbiasedness of the ELL estimator. The bias diagnostic plots with the y = x (dotted) lines and regression (solid) lines shown in Fig 2 (plot (a) for diarrhoea, (c) for ARI and (e) for ARI/diarrhoea) indicate that the ELL estimator provides approximately unbiased estimates compared to the DIR estimates. The bias diagnostic plots indicate that the ELL estimates shrink to the average estimates than the DIR estimates do. The main reason might be high prevalence estimated by the DIR estimator for some districts with small number of children and also zero prevalence (for diarrhoea and ARI) for some districts. For comparing the accuracy of the district-level prevalence estimated by the ELL and the DIR estimators, the CVs of estimated diarrhoea, ARI and ARI/diarrhoea prevalence are plotted against the district index ordered by the number of children in Fig 2 (plots (b), (d), and (f) respectively). The plots for CVs indicate that the ELL estimator provides considerably lower CVs (red triangles) than those of DIR estimator (black circles) as expected. The CVs for DIR estimates are not available for the districts with zero prevalence. This is an advantage of the SAE method, which provides estimates with accuracy for those areas having no information in the sample data. The summary statistics of the district-level prevalence indicate that the diarrhoea prevalence varied within 2.69–8.03%, ARI prevalence within 4.26–8.92%, and ARI/diarrhoea 7.71–13.73% (please see Table 3). Maximum CVs for the district-level prevalence are estimated as 20.81%, 18.25%, and 12.10% respectively, which indicate that the accuracies of the estimated prevalence are reasonably well.

**Table 3:** Summary statistics of the estimated diarrhoea, ARI, and ARI/diarrhoea prevalence among under-5 children at district and sub-district level with their estimated root mean squared errors (RMSE) and coefficient of variation (CV%) using ELL estimator.

Level	Indicator	Statistics	Min	Q1	Mean	Median	Q3	Max	SD
**District**	**Diarrhoea**	Prevalence (%)	2.69	3.79	4.67	4.87	5.69	8.03	1.25
		RMSE × 1000	3.84	4.29	5.15	5.42	6.21	9.02	1.35
		CV (%)	7.97	9.53	10.89	11.41	12.78	20.81	2.50
	**ARI**	Prevalence (%)	4.26	5.44	6.07	6.15	6.74	8.92	0.99
		RMSE × 1000	4.38	5.28	5.78	6.17	6.44	10.64	1.48
		CV (%)	7.51	8.50	9.34	10.16	11.08	18.25	2.46
	**ARI/Diarrhoea**	Prevalence (%)	7.71	9.52	10.32	10.56	11.44	13.73	1.37
		RMSE × 1000	6.16	7.24	7.86	7.96	8.51	11.06	1.05
		CV (%)	5.85	6.85	7.47	7.62	8.20	12.10	1.18
**Sub-district**	**Diarrhoea**	Prevalence (%)	1.38	3.57	4.66	4.91	5.96	11.31	1.70
		RMSE × 1000	3.93	4.80	6.01	7.36	8.36	27.80	3.77
		CV (%)	8.08	11.07	13.32	15.61	16.85	45.35	6.91
	**ARI**	Prevalence (%)	2.21	5.21	6.04	6.05	6.79	11.83	1.34
		RMSE × 1000	4.57	5.91	6.82	7.86	9.04	21.30	2.91
		CV (%)	7.27	9.85	11.36	13.64	14.22	43.33	6.30
	**ARI/Diarrhoea**	Prevalence (%)	4.27	8.96	10.37	10.37	11.85	16.45	2.08
		RMSE × 1000	6.05	7.94	9.14	10.11	11.27	28.25	3.28
		CV (%)	5.66	7.67	8.89	10.17	10.77	28.58	4.05

Since the survey data covers only 396 out of 544 sub-districts and sub-district-specific sample sizes are too small, only the ELL estimator is applied to calculate sub-district-specific prevalence. Summary statistics of the estimated sub-district level prevalence of diarrhoea, ARI and ARI/diarrhoea shown in Table 3 indicate that sub-district level prevalence vary within 1.38–11.31% for diarrhoea, 2.21–11.83% for ARI and 4.27–16.45% for ARI/diarrhoea. It is observed that about 25% sub-districts have more than about 6.0% and 7.0% prevalence of diarrhoea and ARI respectively; while about 75% sub-districts have more than 9.0% prevalence of ARI/diarrhoea. Sub-district level estimates are also efficient based on the CV estimates, of which 75% are below 17% for diarrhoea, 14% for ARI and 11% for ARI/diarrhoea.

**Fig 3:**
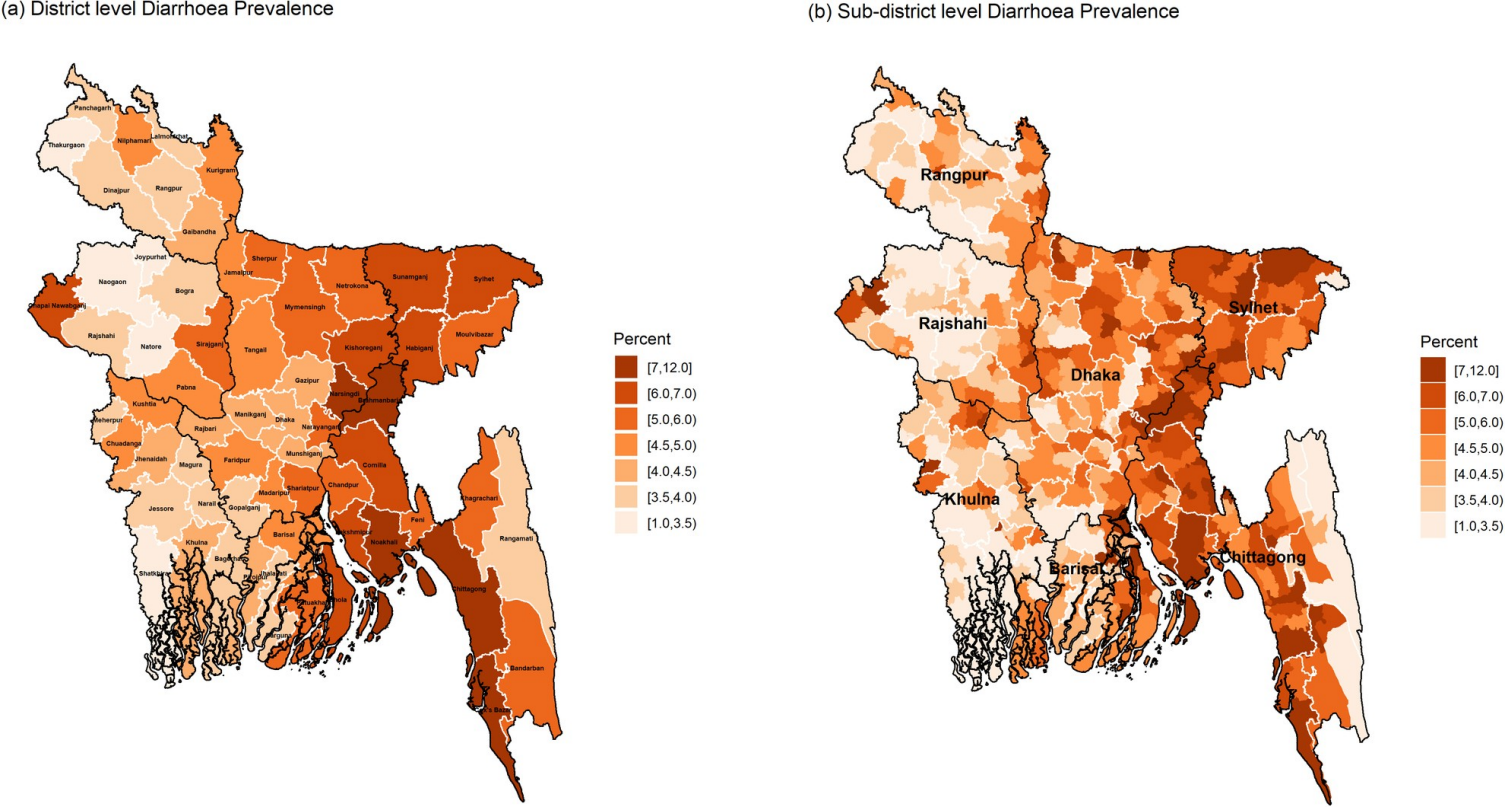
District and sub-district level hotspots of diarrhoea prevalence among under-5 children of Bangladesh (black and white borderlines refer respectively to division and district areas while no border for sub-districts).

For identifying the vulnerable hotspots of the considered three child morbidity indicators, district and sub-district level maps of Bangladesh are generated using the corresponding estimates calculated by the ELL estimator. For each indicator, the maps are constructed using seven-shaded colours based on the same scale for both district and sub-district level maps. Maps in Fig 3 show the spatial distributions of diarrhoea prevalence at district and sub-district levels. District-level map shows that the districts of north-western (*Rangpur* region), western (*Rajshahi* region) and western-south (*Khulna* region) parts have comparatively lower diarrhoea prevalence compared to those districts of northern (*Mymensingh* region), north-eastern (*Sylhet* region), south-eastern (*Chittagong* region) and coastal regions of *Barisal* division. More specifically, the highest diarrhoea prevalence is found in Cox’s Bazar (8%) and the lowest in *Joypurhat* district (3%). Interestingly, “*Chapai Nawabganj*” district lying in the very western part is highly vulnerable to diarrhoea prevalence, though its neighbouring districts are least vulnerable.

The sub-district level map exactly shows the main micro-level hotspots of diarrhoea prevalence. The highly vulnerable sub-districts with a prevalence of more than 7.0% are mostly in the *Sylhet* and *Chittagong* regions, which are highly prone to floods every year. However, it is observed that there are some sub-districts with higher diarrhoea prevalence (say, more than 8%) belong to the districts with lower diarrhoea prevalence (say, less than 5%) in the district-level map (for example *Jiban Nagar* sub-district in *Chuadanga* district). The map clearly shows for which sub-districts, a district is vulnerable to diarrhoea. For example, only two sub-districts of *Chapai Nawabganj* district are the main hotspots for its higher diarrhoea prevalence.

**Fig 4:**
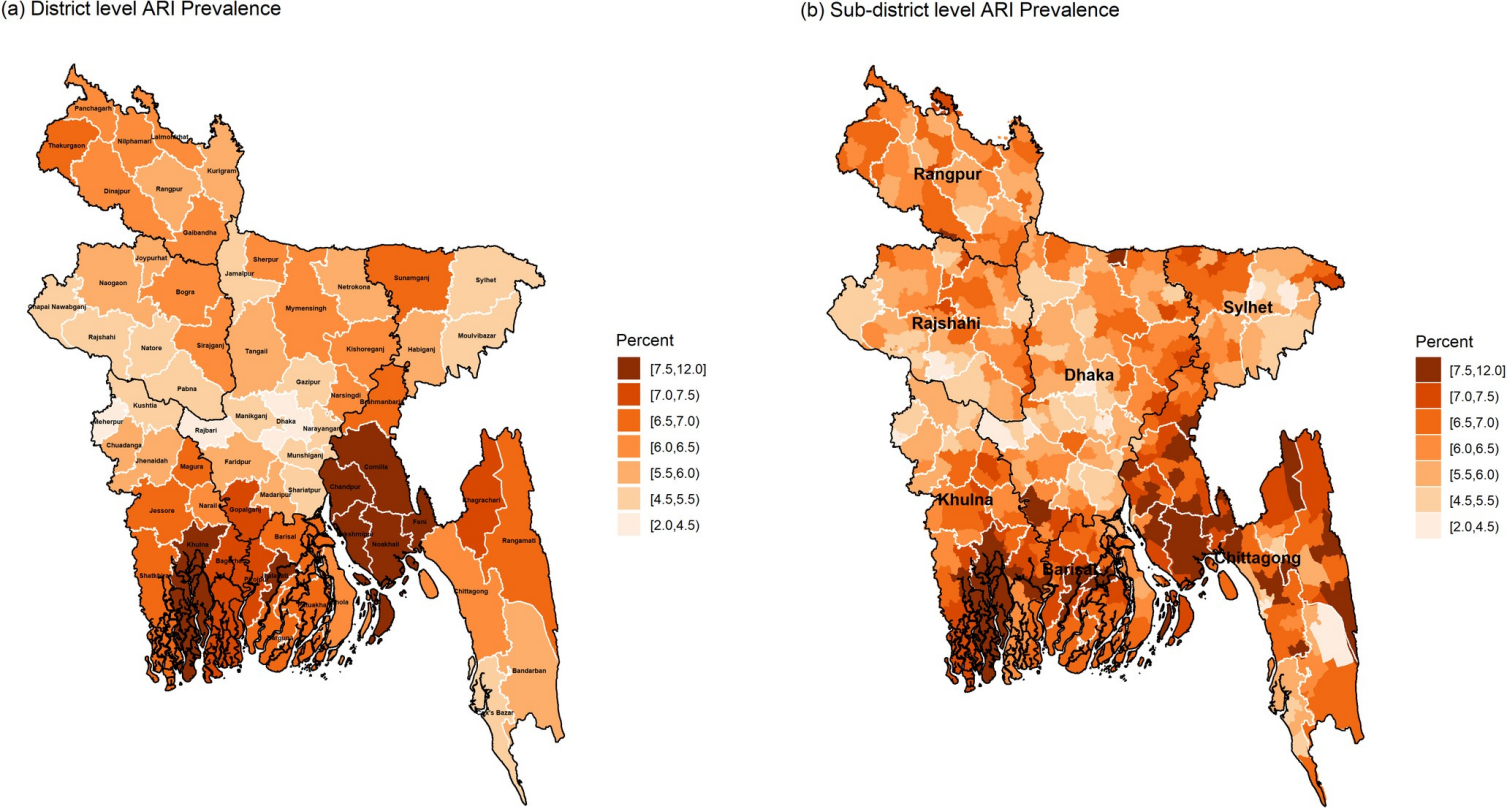
District and sub-district level hotspots of ARI prevalence among under-5 children of Bangladesh (black and white borderlines refer respectively division and district areas while no border for sub-districts).

The district and sub-district level prevalence of ARI are mapped in Fig 4. The district-level map identifies *Chandpur*, *Comilla*, *Feni*, *Noakhali* and *Lakshmipur* districts of *Chittagong* division, *Khulna* district of *Khulna* division and *Jhalokathi* district of *Barisal* division as the highly vulnerable districts for ARI (≥7.5% prevalence). While the sub-district level map shows the hotspots with 7.5–12.0% ARI prevalence not only belonged in the above-mentioned districts but also with other districts lying mostly in the whole coastal southern part of Bangladesh. Also, the children of Hill Tracts area of *Chittagong* division (mainly some sub-districts of *Khagrachari* and *Rangamati* districts) are highly vulnerable to ARI prevalence than to diarrhoea prevalence. Both district and sub-district level maps show hotspots with ARI prevalence of 6.5–7.5% are scattered all over the country except the central region of the country, more specifically the sub-districts surrounding the capital city *Dhaka*.

**Fig 5:**
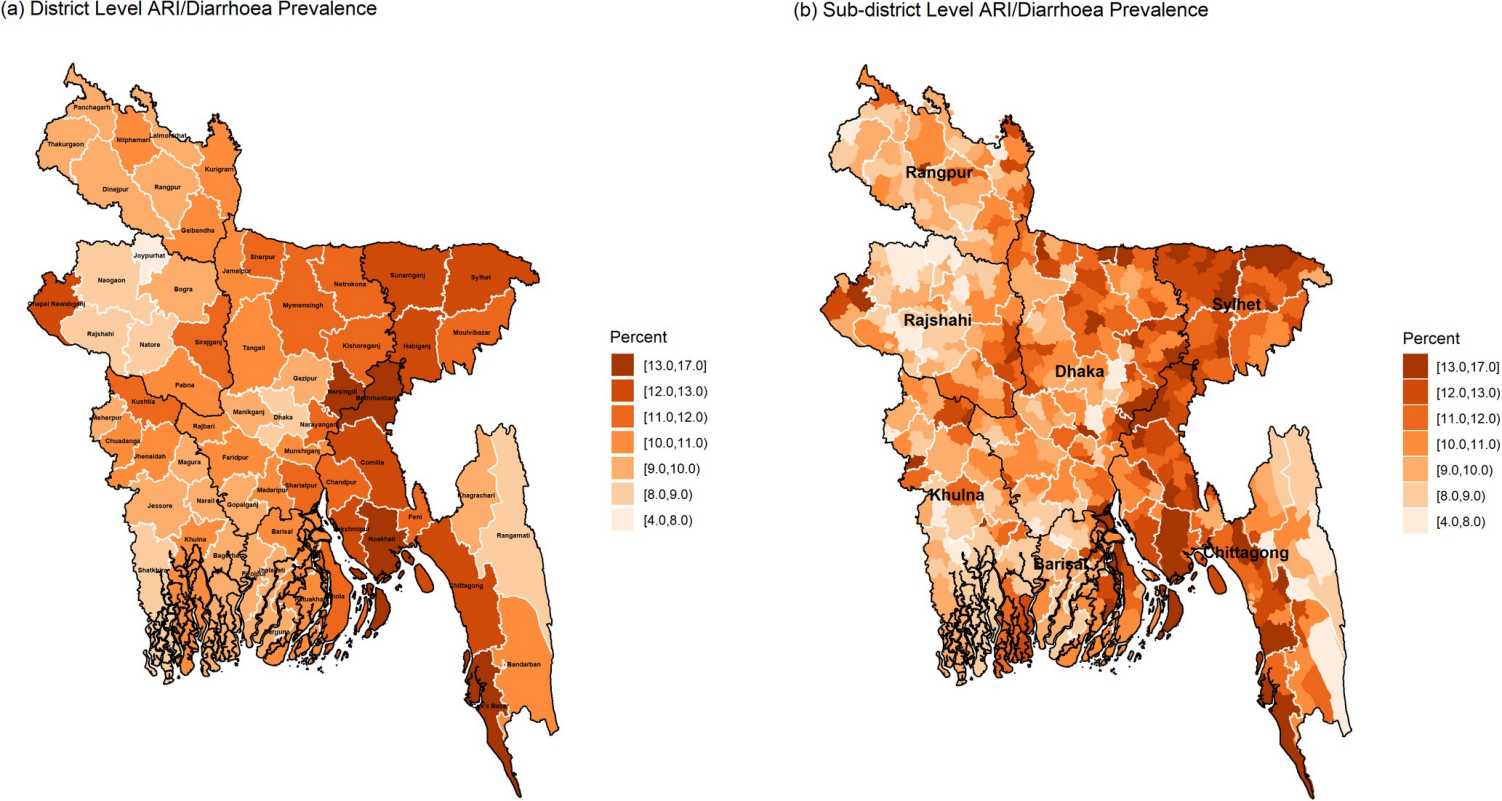
District and sub-district level hotspots of ARI/diarrhoea prevalence among under-5 children of Bangladesh (black and white borderlines refer respectively division and district areas while no border for sub-districts).

The district-level distribution of ARI/Diarrhoea prevalence shown in Fig 5 reveals very similar distribution as for the diarrhoea prevalence. Consequently, there are some tendencies that the sub-districts vulnerable from diarrhoea were also vulnerable from ARI/diarrhoea (such as *Halishahar*, *Teknaf*, *Companiganj*, *Ramu*, and *Ukhia* sub-districts; please see S2 File). The sub-district level map indicates that a significant number of sub-districts particularly from *Sunamganj*, *Sylhet*, *Brahmanbaria*, *Narshingdi*, *Noakhali*, *Chittagong*, *Cox’s Bazar*, *Potuakhali* and *Bhola* districts have more than 12% ARI/diarrhoea prevalence. Overall, the highly vulnerable districts/sub-districts are distributed in the right half of the country except the hill tracts sub-districts (*Khagrachari*, *Rangamati* and *Bandarban* districts) of *Chittagong* region. The children of *Chapai Nawabganj* district along with its two sub-districts (exceptional in the eastern part of the country) are found suffering from both diarrhoea and ARI/diarrhoea but not from ARI separately.

The estimated prevalence of diarrhoea, ARI, and ARI/diarrhoea among under-5 children at district (Zila) and sub-district levels with their 95% CI are given in S1 and S2 Files respectively.

**Fig 6:**
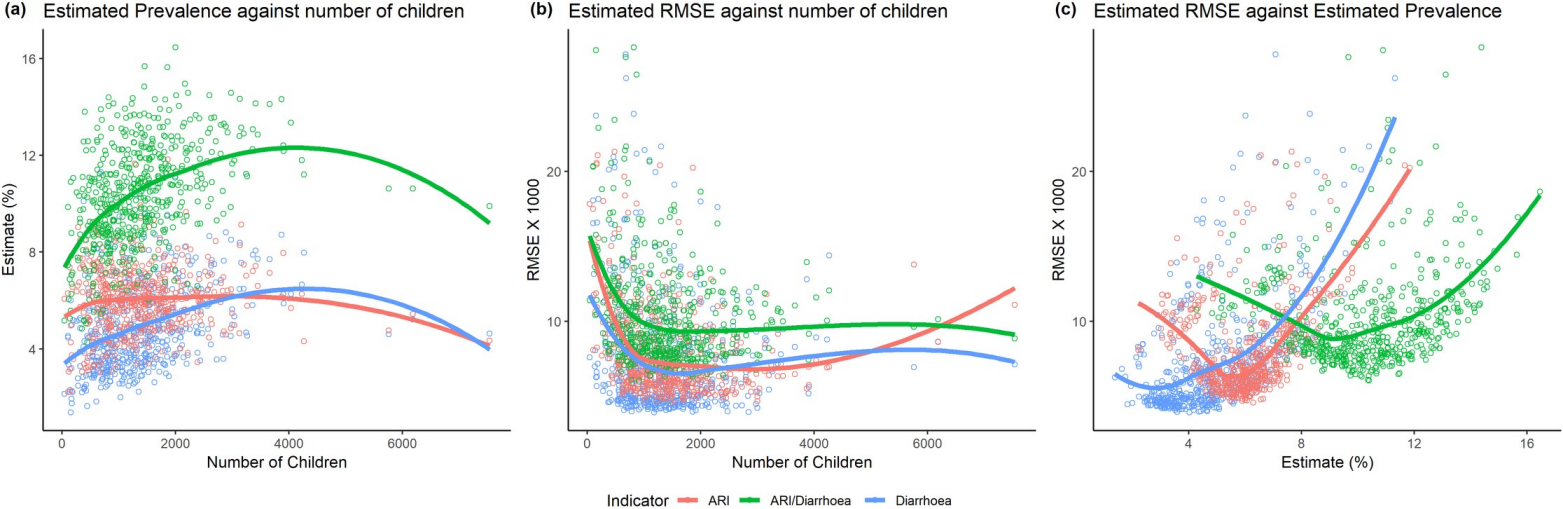
Relationship among the estimated prevalence of diarrhoea, ARI and ARI/diarrhoea among under-5 children at the sub-district level, their standard errors (RMSEX1000), and the sub-district-specific population size (under-5 children).

The characteristics of the ELL estimator for binary response variable are examined by plotting the estimated prevalence and their estimated RMSEs against the sub-district wise total number of children in Fig 6 (plot (a) and (b)). For diarrhoea and ARI/diarrhoea indicators, the estimated prevalence tends to increase exponential with the number of under-5 children (blue and green lines respectively), while the RMSEs are found to have exponential decreasing trend but remains stable for larger sub-districts. For ARI, the smooth line of RMSE (red line) shows an approximate flat U-shape curve with the number of children, however no specific pattern is observed for the estimated prevalence of ARI against the size of the population. The estimated RMSEs are also plotted against the estimated prevalence to examine the ELL estimator (plot (c) in Fig 6). For diarrhoea, the estimated RMSEs increase exponentially with the prevalence, while the smooth lines of ARI and ARI/diarrhoea show approximately U-shape pattern. The U-pattern suggest that the RMSEs decrease quickly up to a prevalence level (near about 5% and 9% for ARI and ARI/diarrhoea respectively) and start to increase after that prevalence level. The distribution of prevalence and their relationship with the number of under-5 children may be one of the reasons for this U-pattern smooth line.

**Fig 7:**
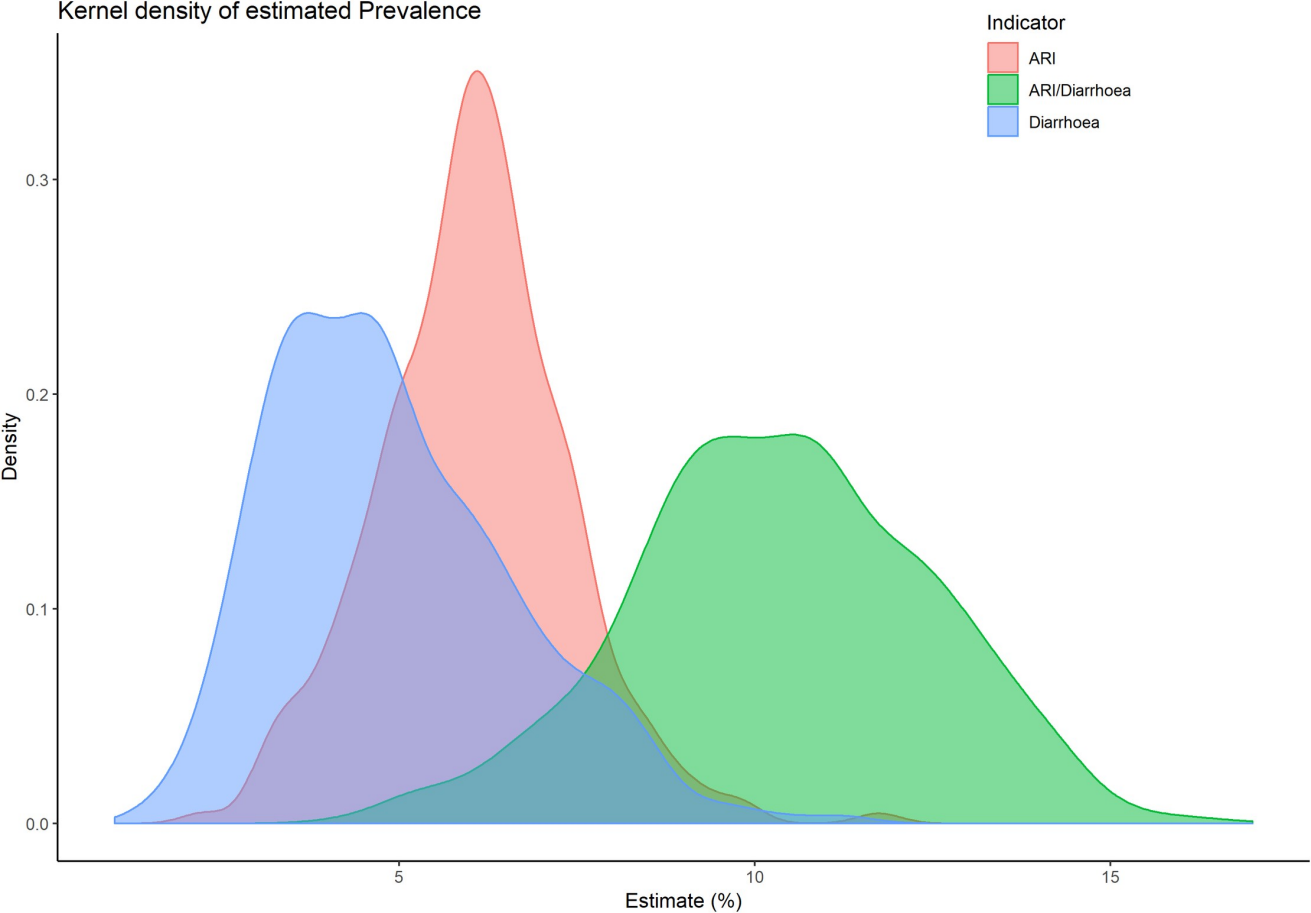
Distribution of sub-district level diarrhoea, ARI, and ARI/diarrhoea prevalence in Bangladesh estimated by the ELL.B estimator.

Kernel densities of the sub-district-specific prevalence of diarrhoea, ARI and ARI/diarrhoea prevalence are plotted in Fig 7. The distribution of diarrhoea prevalence shows slightly positive skewness (blue shaded area), while the distribution of ARI prevalence shows approximately symmetric but a shape of t-distribution with some extreme prevalence on the right tail (red shaded area). On the other hand, the distribution of ARI/diarrhoea shows slightly negative skewness with very flat tail (green shaded area). These skewed distributions indicate that some sub-districts have an unusually high prevalence of diarrhoea (more than 8%) and ARI (more than 10%), while some districts have an unusually lower prevalence of ARI/diarrhoea (less than 5%). Hasslet *et al*. [25] found such positively skewed distribution in the SAE study of diarrhoea prevalence in Nepal.

## Discussion

Diarrhoea and ARI (in the form of pneumonia) are still two leading causes of child deaths not only in Bangladesh but also globally. In Bangladesh, 15% and 6% of 119,000 total under-5 children deaths were due to diarrhoea and pneumonia in 2015 [31]. Delay in seeking appropriate care and lack of access to multiple sources for treatment are recognized as the underlying risk factors for children death due to diarrhoea and pneumonia in Bangladesh [12,13]. Thus, the government should focus on the effective interventions of proper life-saving treatment for the children who are at great risk of pneumonia and diarrhoea to achieve the GDDP goals on reducing child mortality due to ARI and diarrhoea. In this respect, identifying the hotspots of childhood diarrhoea and ARI prevalence at the lower administrative units would be helpful for the government. Taking proper initiative for providing the appropriate life-saving treatments in those hotspots may reduce the mortality due to these two leading causes.

This study aims to identify the hotspots of diarrhoea and ARI prevalence at two disaggregate levels in Bangladesh through the application of an SAE method for the dichotomous response variable. The application provides district and sub-district level prevalence of ARI, diarrhoea, and ARI/diarrhoea episodes with their accuracy measures. The study findings confirmed that though the national and division level estimates of the three health indicators seem very low, there are significant inequalities among districts and sub-districts. The inequality increases when the aggregation level goes down to sub-district from division.

The comparison of the interactive maps of the three indicators at district level suggests that children living in the southern and north-eastern parts of Bangladesh have higher tendency to experience with the prevalence of diarrhoea and ARI related diseases compared to those children living in the central, western and north-western parts. District-level maps indicate that children of five districts (*Sunamganj*, *Brahmanbaria*, *Narshindi*, *Noakhali* and *Cox’s Bazar*) are highly vulnerable to diarrhoea as well as ARI/diarrhoea, while only those of *Noakhali* district are highly susceptible to all the three indicators. The sub-district level maps suggest that the higher risk of occurring child morbidity was not restricted only to those sub-districts belonging to the highly vulnerable districts but also in some districts with less vulnerability. For ARI, moderate to highly vulnerable sub-districts are scattered all over the country with some exception in the central region.

A comparison between district and sub-district level maps helps in identifying those sub-districts for which the prevalence of a specific district becomes higher (for example, three sub-districts of *Chapai Nawabganj* have diarrhoea prevalence of more than 7%, while the other sub-districts have prevalence below 4%). Das, Chandra and Saha [27] also found that *Chapai Nawabganj* had significantly higher diarrhoea prevalence but it was not possible to say for which sub-districts. Sub-district level prevalence of this study indicate that children of “*Gomastapur*”, “*Nawabganj Sadar*” and “*Shibganj*” are more vulnerable to diarrhoea prevalence. It is also observed that some districts with a very low prevalence of an indicator (say, diarrhoea) have sub-districts with considerably higher prevalence (for example, diarrhoea prevalence for *Khagrachari* district is only 5.3% but it has a sub-district (*Manikchari*) with prevalence of 10.1%).

The findings suggest that the ELL estimators for the three dichotomous response variables are providing unbiased and consistent estimates compared to the direct estimator at both district and sub-district level. One of the benefits of this ELL estimator is that an appropriate multilevel model developed at the most detailed level can be used for estimating target parameters at the several higher aggregation levels. In this study, a suitable multilevel model for each of the health indicators has been developed using the survey data and some contextual variables extracted from the 5% census data, and then the prediction has been done using the children level explanatory variables available in the Census data. If full census data can be used, the estimator will be more consistent at the sub-district level and even the indicators can be estimated at lower administrative units like *Union* and *Mauza* (respectively fourth and fifth levels). However, utilizing full census data of Bangladesh will be very tough to handle a mammoth dataset from the perspective of academic researchers. National research institutes like BBS can take initiatives to utilize the full dataset for getting more precise estimates of child morbidity at the most-detailed administrative units.

As limitations of this study, two issues concerned the authors: normality issues of the cluster-specific residuals and the shrinkage of the estimated target parameters to the average. The shrinkage can be removed if more contextual variables at district and sub-district level can be incorporated in the final model. The authors tried to improve the model by incorporating such contextual variables, but the incorporated contextual variables are not found significant in the final model. A bootstrap approach proposed by Carpenter, Goldstein, and Rasbash [32] can be incorporated in the non-parametric bootstrap of ELL method to unshrink the small area estimates. In further studies, the ELL estimator used in this study can be compared to the estimator based on an area-level GLMM model developed at the target aggregation level such as district or sub-district level. For sub-district level, the problem is that about 40% sub-districts are not available in the survey data and so sub-district level random effects cannot be used for prediction for all sub-districts as required in the empirical Bayes estimator described in Molina and Rao [33] and Saei and Chambers [22] utilizing a GLMM model. Ultimately, a synthetic type estimator like ELL will be required for those non-sampled sub-districts.

Though testing the validity of an SAE method is rare due to lack of appropriately detailed data, the ELL method for continuous response variable has been validated by estimating a set of predicted welfare estimates to their true values in the state of Minas Gerais, Brazil [34]. The study findings show that the ELL approach can produce estimates of welfare, which were quite close to their true values (calculated based on census response values). Such kind of validation study for the binary response variable based ELL method used in this study can be conducted in future using only the survey data where any health indicator variable (say, diarrhoea) is available and the survey data structure permits to have sufficient number of observation at a particular lower administrative unit (say district). Das et al. [35] implemented an empirical validation study for the binary response variable based ELL estimator by employing the ELL approach to both height-for-age (HAZ) z-score as continuous response variable and indicator variables (say, stunted: HAZ < -2.00 and severely stunted: < -3.00) by utilizing linear mixed and GLMM models respectively for prediction purpose. Their study findings show that the ELL-type estimator for the binary response variable provides reasonably consistent estimates as the continuous response variable based standard ELL method. However, finding a good GLMM model for the indicator like diarrhoea and ARI is tough due to lack of proper explanatory variables in the census data. This is also a major problem for the area-level model experienced in recent SAE study on diarrhoea prevalence in Bangladesh [27]. As for example, statistics on handwashing (a major determinant of diarrhoea) during diarrhoeal episodes in children and adults cannot be extracted from any administrative source. In addition, the time gap between survey and census year for implementing an SAE study could be an important issue if there are not enough time-invariant variables available in the census data [36].

Children malnutrition status is highly correlated with their recent experience with the occurrence of diarrhoea and ARI related diseases. Evidence from several child malnutrition studies [37, 38] suggest that the children recently suffered from diarrhoea and ARI are more likely to be either wasted (an indicator of acute malnutrition) or underweight (combination of acute and chronic malnutrition). The national target of reducing wasting at 5% by 2025 [39] will be achievable if the prevalence of ARI and diarrhoea can be reduced as well. Haslett, Jones and Isidro [21] conducted a small area study on child undernutrition (underweight: lower weight compared to age) in Bangladesh using the Child and Mother Nutrition Survey of Bangladesh 2012 and full data of Census 2011. They found that the children living in the north-eastern (*Sylhet* and *Mymensingh* regions) and the south-eastern (costal parts of *Chittagong* region) parts of Bangladesh are vulnerable to both underweight and severely underweight (Appendix D.3 of Haslett, Jones and Isidro [21]). These spatial distributions of underweight and severe underweight seem comparable to the spatial distribution of ARI/diarrhoea generated in this study (map (b) in Fig 5). The comparison of these spatial distributions reveals the inter-relationship between child undernutrition and the occurrence of recent diarrhoea and ARI. Thus, the combination of this small area study on child diarrhoea and ARI prevalence with the study of child undernutrition [21] might guide the stakeholders for taking proper initiatives at the lower administrative levels for reducing the vulnerability of child morbidity and undernutrition simultaneously. The interventions at the lower administrative units can be followed up so that the children in risk of severe diarrhoea and pneumonia can get the access to life-saving treatment timely, which save the children as well as reduce the rate of child mortality due to diarrhoea and ARI.

## Conclusions

Disaggregated level prevalence of ARI, diarrhoea, and ARI/diarrhoea within a period of two-week preceding the survey among under-5 Bangladeshi children are estimated in this study through the implementation of an SAE method for the binary outcome variable. The World Bank ELL method has been adapted for a binary response variable by developing a random-effects logistic model for each of the indicators. The estimated prevalence of each indicator significantly vary at the considered district and sub-district levels; even the proportions are found double the national level in some sub-districts. Spatial distributions of the indicators indicate that children of southern and north-eastern regions are more susceptible to be experienced with the occurrence of diarrhoea and ARI. The most disaggregated level maps suggest that vulnerable sub-districts spread over both highly and less vulnerable aggregated administrative units (say division and district), particularly higher prevalence of ARI are observed in all regions except some sub-districts close to the capital city *Dhaka*. These disaggregated level statistics on ARI and diarrhoea prevalence might help the policymakers to identify the susceptible hotspots, which in turn help the aid industries in effective interventions at the highly vulnerable disaggregated administrative units.

## Supporting information

S1 Fig Normal Q-Q plots and distributions of the cluster-specific residuals for the fitted models of diarrhoea, ARI and ARI/diarrhoea. (TIF)

S1 File Estimated prevalence of diarrhoea, ARI, and ARI/diarrhoea among under-5 children at district (Zila) level with their 95% CI. (XLSX)

S2 File Estimated prevalence of diarrhoea, ARI, and ARI/diarrhoea among under-5 children at sub-district (Up-zila) level with their 95% CI. (XLSX)

S1 Table Regression Coefficients (p-values) of the fitted model for diarrhoea, ARI and ARI/diarrhoea based on BDHS 2011 and Census 2011 data. (DOCX)

S2 Table Description of the contextual variables at different levels calculated from the 5% of Census 2011 data. (DOCX)

S1 Data (ZIP)
