# Supplementary figures and images for: Disaggregated level child morbidity in Bangladesh: An application of small area estimation method
Source: PLoS One. 2020 May 20;15(5):e0220164. doi: 10.1371/journal.pone.0220164 (PMC7239471; doi:10.1371/journal.pone.0220164)

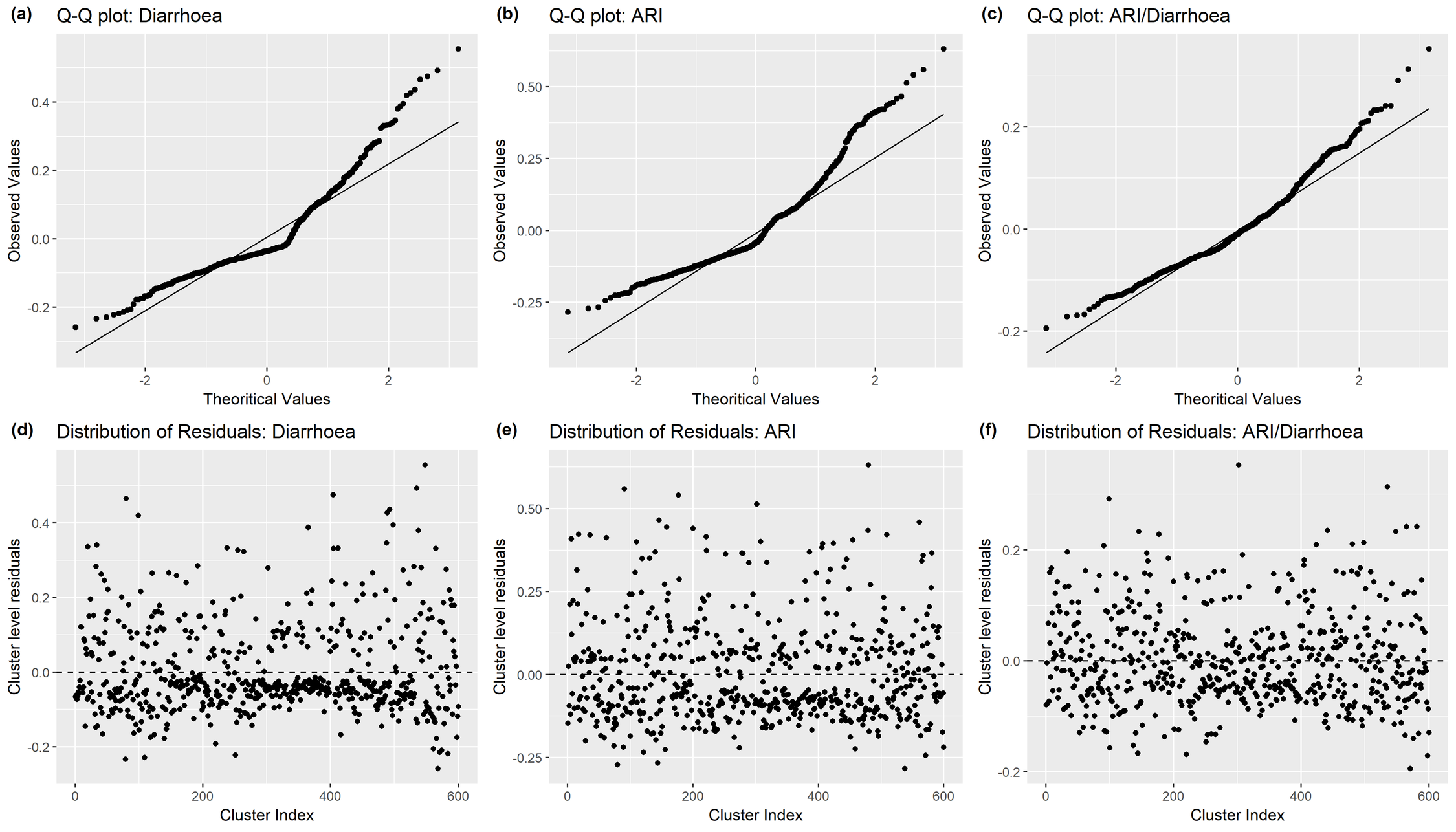

Supplement: S1 Fig — (TIF) [file pone.0220164.s001.tif]
