# Supplementary material for: Disaggregated level child morbidity in Bangladesh: An application of small area estimation method
Source: PLoS One. 2020 May 20;15(5):e0220164. doi: 10.1371/journal.pone.0220164 (PMC7239471; doi:10.1371/journal.pone.0220164)
Supplement: S1 Table — (DOCX) [file pone.0220164.s004.docx]

**S1 Table. Regression Coefficients (p-values) of the fitted model for diarrhoea, ARI and ARI/diarrhoea based on BDHS 2011 and Census 2011 data**

| **Explanatory**  **Variables** | **Diarrhoea** | | **ARI** | | **ARI/Diarrhoea** | |
| --- | --- | --- | --- | --- | --- | --- |
|  | $\hat{\boldsymbol{\beta}}$ | **p-value** | $\hat{\boldsymbol{\beta}}$ | **p-value** | $\hat{\boldsymbol{\beta}}$ | **p-value** |
| (Intercept) | -3.408 | 0.002 | -2.158 | 0.000 | -2.827 | 0.000 |
| Age 1 | 0.164 | 0.330 |  |  |  |  |
| Age 2 | -0.523 | 0.009 |  |  |  |  |
| Age 3 | -0.498 | 0.007 |  |  |  |  |
| Age 4 | -0.832 | 0.000 |  |  |  |  |
| %literate |  |  |  |  | -0.435 | 0.018 |
| %kids714 |  |  | -0.808 | 0.021 |  |  |
| Urban | 0.837 | 0.081 |  |  |  |  |
| Urban:hfem | 1.342 | 0.033 |  |  |  |  |
| Urban:pliterate | -1.192 | 0.014 |  |  |  |  |
| Male: AGE 0 |  |  | 0.877 | 0.000 | 0.826 | 0.000 |
| Female: AGE 0 |  |  | 0.434 | 0.081 | 0.440 | 0.017 |
| Male: AGE 1 |  |  | 0.918 | 0.000 | 0.945 | 0.000 |
| Female: AGE 1 |  |  | 0.602 | 0.013 | 0.685 | 0.000 |
| Male: AGE 2 |  |  | 0.585 | 0.018 | 0.519 | 0.005 |
| Female: AGE 2 |  |  | 0.596 | 0.014 | 0.421 | 0.023 |
| Male: AGE 3 |  |  | 0.456 | 0.057 | 0.324 | 0.072 |
| Female: AGE 3 |  |  | 0.297 | 0.239 | 0.123 | 0.522 |
| Male: AGE 4 |  |  | 0.494 | 0.042 | 0.143 | 0.451 |
| Urban: Age 1 | 0.263 | 0.441 |  |  |  |  |
| Urban: Age 2 | 0.758 | 0.039 |  |  |  |  |
| Urban: Age 3 | -0.076 | 0.854 |  |  |  |  |
| Urban: Age 4 | 0.451 | 0.270 |  |  |  |  |
| Urban: *Barisal* |  |  | -0.167 | 0.580 | 0.150 | 0.497 |
| Urban: *Chittagong* | -0.490 | 0.167 | -0.425 | 0.080 | -0.218 | 0.213 |
| Urban: *Dhaka* | -1.127 | 0.005 | -0.299 | 0.325 | -0.691 | 0.001 |
| Urban: *Khulna* | -0.826 | 0.066 | 0.115 | 0.656 | -0.023 | 0.912 |
| Urban: *Rajshahi* | -0.902 | 0.047 | 0.074 | 0.790 | -0.199 | 0.370 |
| Urban: *Rangpur* | -0.958 | 0.044 | 0.088 | 0.749 | -0.085 | 0.704 |
| Urban: *Sylhet* | -0.557 | 0.155 | -1.026 | 0.008 | -0.502 | 0.027 |
| **Sub-district Level Contextual Variables** | | | | | | |
| %emp15_subdist |  |  | -2.865 | 0.043 |  |  |
| %lit_subdist | -2.832 | 0.000 | -9.018 | 0.005 | -1.239 | 0.025 |
| %hhlit_subdist |  |  | 6.056 | 0.003 |  |  |
| %hhcssc_subdist |  |  | 3.985 | 0.081 |  |  |
| %empag_subdist | -5.112 | 0.000 |  |  |  |  |
| %fcmced_subdist | 2.628 | 0.005 |  |  | 1.440 | 0.027 |
| %adnowork_subdist |  |  |  |  | -3.316 | 0.000 |
