# Supplementary material for: Disaggregated level child morbidity in Bangladesh: An application of small area estimation method
Source: PLoS One. 2020 May 20;15(5):e0220164. doi: 10.1371/journal.pone.0220164 (PMC7239471; doi:10.1371/journal.pone.0220164)
Supplement: S2 Table — (DOCX) [file pone.0220164.s005.docx]

**S2 Table. Description of the contextual variables at different levels calculated from the 5% of Census 2011 data**

| **Name** | **Description** | **Type** |
| --- | --- | --- |
| hfem | Is the household (HH) head female? | Indicator |
| %literate | Proportion of literate people in HH | Continuous |
| %kids714 | proportion of HH member aged 7-14 years | Continuous |
| **Sub-district Level Variables** | | |
| %emp15_subdist | Proportion of 15+ persons employed | Continuous |
| %lit_subdist | Proportion of 7+persons can write a letter | Continuous |
| %hhlit_subdist | Proportion of HH member with literate head | Continuous |
| %hhcssc_subdist | Proportion of HH with at least one secondary educated member | Continuous |
| %adnowork_subdist | Proportion of unemployed adults | Continuous |
| %fcmced_subdist | Ratio of female children to male children in primary school | Continuous |
| %empag_subdist | Proportion of 15+ persons employed in agriculture | Continuous |

Source: Author’s calculation
