# Supplementary material for: Disaggregated level child morbidity in Bangladesh: An application of small area estimation method
Source: PLoS One. 2020 May 20;15(5):e0220164. doi: 10.1371/journal.pone.0220164 (PMC7239471; doi:10.1371/journal.pone.0220164)
Supplement: Attachment — Submitted filename: Review_SEA_Model_Child_Morbidity_Nov2019.docx [file pone.0220164.s007.docx]

Review of “Disaggregated Level Child Morbidity in Bangladesh: An Application of Small Area Estimation Method.”

This is an interesting paper about the application of a small area estimation (SEA) method to child morbidity in Bangladesh. The paper is useful as it extends a technique used by the World Bank for continuous variable (consumption per capita and nutrition Z-score) to dichotomous variables on ARI and diarrhea. The authors apply the method to 2011 data from the BDHS and Bangladesh census.

A few comments:

- It will be good to present more information on applications of SEA to other countries, even if using continuous variable. Particular emphasis could be discussing any validation studies or comparisons of the small area estimates obtained with SEA with estimates obtained from other sources for the small areas or with estimates that came from a census (or sample from a census).
- The authors make a good argumentation on the fitness of the model, however, the figures were barely readable. For a method based largely on figures, it was not good.
- In page 15, the authors discuss Table 3. The text says “Table 3 indicates that the mean …” but the estimates presented are from the “Median” column in Table 3.
- This application had the benefit of having the DHS and the census sample from the year. It will be good for the authors to elaborate on the application of SEA in other situations of data availability that are more prevalent in developing countries, such as, a number of years of separation between the DHS and a census (an its sample), or having a limited number of common variables in the two data sources.
