# Supplementary material for: Disaggregated level child morbidity in Bangladesh: An application of small area estimation method
Source: PLoS One. 2020 May 20;15(5):e0220164. doi: 10.1371/journal.pone.0220164 (PMC7239471; doi:10.1371/journal.pone.0220164)
Supplement: Peer review history

# Peer review: Decision Letter 0

**DOI:** 10.1371/journal.pone.0220164.r001

**Authors:** Tabassum Insaf

21 Nov 2019

PONE-D-19-18946

Disaggregated Level Child Morbidity in Bangladesh: An Application of Small Area Estimation Method

PLOS ONE

Dear Dr. Kawsar,

Thank you for submitting your manuscript to PLOS ONE. After careful consideration, we feel that it has merit but does not fully meet PLOS ONE’s publication criteria as it currently stands. Therefore, we invite you to submit a revised version of the manuscript that addresses the points raised during the review process.

==============================

ACADEMIC EDITOR: Please insert comments here and delete this placeholder text when finished. Be sure to:

- Indicate which changes are required versus recommended for acceptance
- Address any conflicts between the reviews
- Provide specific feedback from your evaluation of the manuscript

==============================

We would appreciate receiving your revised manuscript by Jan 05 2020 11:59PM. When you are ready to submit your revision, log on to https://www.editorialmanager.com/pone/ and select the 'Submissions Needing Revision' folder to locate your manuscript file.

If you would like to make changes to your financial disclosure, please include your updated statement in your cover letter.

To enhance the reproducibility of your results, we recommend that if applicable you deposit your laboratory protocols in protocols.io, where a protocol can be assigned its own identifier (DOI) such that it can be cited independently in the future. For instructions see: http://journals.plos.org/plosone/s/submission-guidelines#loc-laboratory-protocols

Please include the following items when submitting your revised manuscript:

- A rebuttal letter that responds to each point raised by the academic editor and reviewer(s). This letter should be uploaded as separate file and labeled 'Response to Reviewers'.
- A marked-up copy of your manuscript that highlights changes made to the original version. This file should be uploaded as separate file and labeled 'Revised Manuscript with Track Changes'.
- An unmarked version of your revised paper without tracked changes. This file should be uploaded as separate file and labeled 'Manuscript'.

Please note while forming your response, if your article is accepted, you may have the opportunity to make the peer review history publicly available. The record will include editor decision letters (with reviews) and your responses to reviewer comments. If eligible, we will contact you to opt in or out.

We look forward to receiving your revised manuscript.

Kind regards,

Tabassum Insaf

Academic Editor

PLOS ONE

Journal Requirements:

When submitting your revision, we need you to address these additional requirements.

Please ensure that your manuscript meets PLOS ONE's style requirements, including those for file naming. The PLOS ONE style templates can be found at

http://www.journals.plos.org/plosone/s/file?id=wjVg/PLOSOne_formatting_sample_main_body.pdf and http://www.journals.plos.org/plosone/s/file?id=ba62/PLOSOne_formatting_sample_title_authors_affiliations.pdf

1. Our internal editors have looked over your manuscript and determined that it may be within the scope of our Mathematical Modelling of Infectious Disease Dynamics Call for Papers. The Collection will encompass a diverse range of research articles on using mathematical models to better understand infectious diseases. Additional information can be found on our announcement page: https://collections.plos.org/s/mathematical-disease-dynamics. If you would like your manuscript to be considered for this collection, please let us know in your cover letter and we will ensure that your paper is treated as if you were responding to this call. If you would prefer to remove your manuscript from collection consideration, please specify this in the cover letter.

2. Please note that PLOS ONE has specific guidelines on software sharing (http://journals.plos.org/plosone/s/materials-and-software-sharing#loc-sharing-software) for manuscripts whose main purpose is the description of a new software or software package. In this case, new software must conform to the Open Source Definition (https://opensource.org/docs/osd) and be deposited in an open software archive. Please see http://journals.plos.org/plosone/s/materials-and-software-sharing#loc-depositing-software for more information on depositing your software.

3. We note that you have stated that you will provide repository information for your data at acceptance. Should your manuscript be accepted for publication, we will hold it until you provide the relevant accession numbers or DOIs necessary to access your data. If you wish to make changes to your Data Availability statement, please describe these changes in your cover letter and we will update your Data Availability statement to reflect the information you provide.

4. Please amend either the title on the online submission form (via Edit Submission) or the title in the manuscript so that they are identical.

5. We note that [Figure(s) 4,5,6] in your submission contain [map/satellite] images which may be copyrighted. All PLOS content is published under the Creative Commons Attribution License (CC BY 4.0), which means that the manuscript, images, and Supporting Information files will be freely available online, and any third party is permitted to access, download, copy, distribute, and use these materials in any way, even commercially, with proper attribution. For these reasons, we cannot publish previously copyrighted maps or satellite images created using proprietary data, such as Google software (Google Maps, Street View, and Earth). For more information, see our copyright guidelines: http://journals.plos.org/plosone/s/licenses-and-copyright.

We require you to either (1) present written permission from the copyright holder to publish these figures specifically under the CC BY 4.0 license, or (2) remove the figures from your submission:

1.    You may seek permission from the original copyright holder of Figure(s) [4,5,6] to publish the content specifically under the CC BY 4.0 license. 

We recommend that you contact the original copyright holder with the Content Permission Form (http://journals.plos.org/plosone/s/file?id=7c09/content-permission-form.pdf) and the following text:

“I request permission for the open-access journal PLOS ONE to publish XXX under the Creative Commons Attribution License (CCAL) CC BY 4.0 (http://creativecommons.org/licenses/by/4.0/). Please be aware that this license allows unrestricted use and distribution, even commercially, by third parties. Please reply and provide explicit written permission to publish XXX under a CC BY license and complete the attached form.”

Please upload the completed Content Permission Form or other proof of granted permissions as an "Other" file with your submission.

In the figure caption of the copyrighted figure, please include the following text: “Reprinted from [ref] under a CC BY license, with permission from [name of publisher], original copyright [original copyright year].”

2.    If you are unable to obtain permission from the original copyright holder to publish these figures under the CC BY 4.0 license or if the copyright holder’s requirements are incompatible with the CC BY 4.0 license, please either i) remove the figure or ii) supply a replacement figure that complies with the CC BY 4.0 license. Please check copyright information on all replacement figures and update the figure caption with source information. If applicable, please specify in the figure caption text when a figure is similar but not identical to the original image and is therefore for illustrative purposes only.

The following resources for replacing copyrighted map figures may be helpful:

USGS National Map Viewer (public domain): http://viewer.nationalmap.gov/viewer/

The Gateway to Astronaut Photography of Earth (public domain): http://eol.jsc.nasa.gov/sseop/clickmap/

Maps at the CIA (public domain): https://www.cia.gov/library/publications/the-world-factbook/index.html and https://www.cia.gov/library/publications/cia-maps-publications/index.html

NASA Earth Observatory (public domain): http://earthobservatory.nasa.gov/

Landsat: http://landsat.visibleearth.nasa.gov/

USGS EROS (Earth Resources Observatory and Science (EROS) Center) (public domain): http://eros.usgs.gov/#

Natural Earth (public domain): http://www.naturalearthdata.com/

[Note: HTML markup is below. Please do not edit.]

Reviewers' comments:

Reviewer's Responses to Questions

**Comments to the Author**

1. Is the manuscript technically sound, and do the data support the conclusions?

The manuscript must describe a technically sound piece of scientific research with data that supports the conclusions. Experiments must have been conducted rigorously, with appropriate controls, replication, and sample sizes. The conclusions must be drawn appropriately based on the data presented. 

Reviewer #1: Yes

Reviewer #2: Yes

**********

2. Has the statistical analysis been performed appropriately and rigorously? 

Reviewer #1: Yes

Reviewer #2: Yes

**********

3. Have the authors made all data underlying the findings in their manuscript fully available?

The PLOS Data policy requires authors to make all data underlying the findings described in their manuscript fully available without restriction, with rare exception (please refer to the Data Availability Statement in the manuscript PDF file). The data should be provided as part of the manuscript or its supporting information, or deposited to a public repository. For example, in addition to summary statistics, the data points behind means, medians and variance measures should be available. If there are restrictions on publicly sharing data—e.g. participant privacy or use of data from a third party—those must be specified.

Reviewer #1: No

Reviewer #2: No

**********

4. Is the manuscript presented in an intelligible fashion and written in standard English?

PLOS ONE does not copyedit accepted manuscripts, so the language in submitted articles must be clear, correct, and unambiguous. Any typographical or grammatical errors should be corrected at revision, so please note any specific errors here.

Reviewer #1: Yes

Reviewer #2: Yes

**********

5. Review Comments to the Author

Please use the space provided to explain your answers to the questions above. You may also include additional comments for the author, including concerns about dual publication, research ethics, or publication ethics. (Please upload your review as an attachment if it exceeds 20,000 characters)

Reviewer #1: p8, I would avoid using language that refers to the outcomes as "event" as it implies these are incident rates, these are from a survey so they are prevalences, and the event language should not be used. I would suggest using the term "outcome"

What software was used to do the analysis?

Where are the sub-district level variables derived from? the DHS itself? specify source and if you derived them, how this was done

In appendix table 1, instead of pliterate and other variable names from the computer software, spell these out (e.g. % literate), same with the sub-district level variable names

I don't think the figure 1 is necessary

I think the figures in figure 3 on the right would be better presented as points,vs the line, also i'm not sure from the figure what the solid black line represents

Figure 4, remove the blue lines between districts, use a thin black line instead. Also the islands on the ocean side of the country are basically illegible because there are so many, would simplified shapefile be a better choice for this presentation?

Have the district and sub-district maps have the same scales so the colors are comparable between the two levels.

I would recommend a formally defined Discussion and Conclusion section of the manuscript

Grammatical notes

p5. change area level method to area level methods

p5. change due to zero observation to due to zero observations

p5. change however not possible to estimate to however it is not possible to estimate

p7. change source of drinking water, sanitation to source of drinking water, and household sanitation

p8, multilevel is misspelled as multilvel

p8. problematic is misspelled as problemtic

p8 change discussed in model development section to discussed in the model development section

Reviewer #2: This is an interesting paper about the application of a small area estimation (SEA) method to child morbidity in Bangladesh. The paper is useful as it extends a technique used by the World Bank for continuous variable (consumption per capita and nutrition Z-score) to dichotomous variables on ARI and diarrhea. The authors apply the method to 2011 data from the BDHS and Bangladesh census.

A few comments:

- It will be good to present more information on applications of SEA to other countries, even if using continuous variable. Particular emphasis could be discussing any validation studies or comparisons of the small area estimates obtained with SEA with estimates obtained from other sources for the small areas or with estimates that came from a census (or sample from a census).

- The authors make a good argumentation on the fitness of the model, however, the figures were barely readable. For a method based largely on figures, it was not good.

- In page 15, the authors discuss Table 3. The text says “Table 3 indicates that the mean …” but the estimates presented are from the “Median” column in Table 3.

- This application had the benefit of having the DHS and the census sample from the year. It will be good for the authors to elaborate on the application of SEA in other situations of data availability that are more prevalent in developing countries, such as, a number of years of separation between the DHS and a census (an its sample), or having a limited number of common variables in the two data sources.

**********

6. PLOS authors have the option to publish the peer review history of their article (what does this mean?). If published, this will include your full peer review and any attached files.

If you choose “no”, your identity will remain anonymous but your review may still be made public.

**Do you want your identity to be public for this peer review?** For information about this choice, including consent withdrawal, please see our Privacy Policy.

Reviewer #1: No

Reviewer #2: No

[NOTE: If reviewer comments were submitted as an attachment file, they will be attached to this email and accessible via the submission site. Please log into your account, locate the manuscript record, and check for the action link "View Attachments". If this link does not appear, there are no attachment files to be viewed.]

While revising your submission, please upload your figure files to the Preflight Analysis and Conversion Engine (PACE) digital diagnostic tool, https://pacev2.apexcovantage.com/. PACE helps ensure that figures meet PLOS requirements. To use PACE, you must first register as a user. Registration is free. Then, login and navigate to the UPLOAD tab, where you will find detailed instructions on how to use the tool. If you encounter any issues or have any questions when using PACE, please email us at figures@plos.org. Please note that Supporting Information files do not need this step.

Attachment Submitted filename: Review_SEA_Model_Child_Morbidity_Nov2019.docx Click here for additional data file.

# Author comment: Author response to Decision Letter 0

**DOI:** 10.1371/journal.pone.0220164.r002

3 Jan 2020

Rebuttal letter

1. Our internal editors have looked over your manuscript and determined that it may be within the scope of our Mathematical Modelling of Infectious Disease Dynamics Call for Papers. The Collection will encompass a diverse range of research articles on using mathematical models to better understand infectious diseases. Additional information can be found on our announcement page: https://collections.plos.org/s/mathematical-disease-dynamics. If you would like your manuscript to be considered for this collection, please let us know in your cover letter and we will ensure that your paper is treated as if you were responding to this call. If you would prefer to remove your manuscript from collection consideration, please specify this in the cover letter.

Response: We are happy to publish this work under the Mathematical Modelling of Infectious Disease Dynamics Call for Papers. So please assign the revised version of the paper for the special issue.

2. Please note that PLOS ONE has specific guidelines on software sharing (http://journals.plos.org/plosone/s/materials-and-software-sharing#loc-sharing-software) for manuscripts whose main purpose is the description of a new software or software package. In this case, new software must conform to the Open Source Definition (https://opensource.org/docs/osd) and be deposited in an open software archive. Please see http://journals.plos.org/plosone/s/materials-and-software-sharing#loc-depositing-software for more information on depositing your software.

Response: We did not develop any software. We used some R packages to analyze the data.

3. We note that you have stated that you will provide repository information for your data at acceptance. Should your manuscript be accepted for publication, we will hold it until you provide the relevant accession numbers or DOIs necessary to access your data. If you wish to make changes to your Data Availability statement, please describe these changes in your cover letter and we will update your Data Availability statement to reflect the information you provide.

Response: We have attached the used survey data (BDHS 2011) which are available freely from DHS.com. We have collected the 5% of the Census data for academic purpose from Bangladesh Bureau of Statistics. Someone can contact with the department of Micro data & Metadata Information (http://203.112.218.65:8008/PageWebMenuContent.aspx?MenuKey=62). The 5% census data of Bangladesh is now freely available upon request from Integrated Public Use Microdata Series (IPUMS) International (https://international.ipums.org/international/) who provides harmonized international census data for social science and health research. The “Minimal Dataset” used for producing the results of this paper are given as supplementary files as csv format.

4. Please amend either the title on the online submission form (via Edit Submission) or the title in the manuscript so that they are identical.

Response: We wish to keep the title of the paper same as initial submission.

5. We note that [Figure(s) 4,5,6] in your submission contain [map/satellite] images which may be copyrighted. All PLOS content is published under the Creative Commons Attribution License (CC BY 4.0), which means that the manuscript, images, and Supporting Information files will be freely available online, and any third party is permitted to access, download, copy, distribute, and use these materials in any way, even commercially, with proper attribution. For these reasons, we cannot publish previously copyrighted maps or satellite images created using proprietary data, such as Google software (Google Maps, Street View, and Earth). For more information, see our copyright guidelines: http://journals.plos.org/plosone/s/licenses-and-copyright.

Response: We want to confirm you that the Bangladesh maps are created using R packages (sp and sf) using the shape files available in https://gadm.org/download_country_v3.html online. The shape files are also available from the website of the concerned Ministry of Bangladesh. So we think, the generated maps are fine to publish. Someone can easily download the shape-files from the above-mentioned website and can produce the same types of Bangladesh maps at district and sub-district levels. 

Comments to the Author

1. Is the manuscript technically sound, and do the data support the conclusions?

The manuscript must describe a technically sound piece of scientific research with data that supports the conclusions. Experiments must have been conducted rigorously, with appropriate controls, replication, and sample sizes. The conclusions must be drawn appropriately based on the data presented.

Reviewer #1: Yes

Reviewer #2: Yes

2. Has the statistical analysis been performed appropriately and rigorously?

Reviewer #1: Yes

Reviewer #2: Yes

3. Have the authors made all data underlying the findings in their manuscript fully available?

The PLOS Data policy requires authors to make all data underlying the findings described in their manuscript fully available without restriction, with rare exception (please refer to the Data Availability Statement in the manuscript PDF file). The data should be provided as part of the manuscript or its supporting information, or deposited to a public repository. For example, in addition to summary statistics, the data points behind means, medians and variance measures should be available. If there are restrictions on publicly sharing data—e.g. participant privacy or use of data from a third party—those must be specified.

Reviewer #1: No

Reviewer #2: No

Response: We have provided “Minimal Dataset” of Survey and the census (Minimal Dataset Plos One.zip) from which the results of the paper are generated. The sources of data are given in the above response.

4. Is the manuscript presented in an intelligible fashion and written in standard English?

PLOS ONE does not copyedit accepted manuscripts, so the language in submitted articles must be clear, correct, and unambiguous. Any typographical or grammatical errors should be corrected at revision, so please note any specific errors here.

Reviewer #1: Yes

Reviewer #2: Yes

Response: Thanks for the comment.

5. Review Comments to the Author

Reviewer #1: 

p8, I would avoid using language that refers to the outcomes as "event" as it implies these are incident rates, these are from a survey so they are prevalences, and the event language should not be used. I would suggest using the term "outcome"

Response: Thanks for bring out the issue of incidence and prevalence. We did not use the “event” term for calculating incidence rate. Since there may have some confusion, we have revised the “event” term by the recommended “outcome” term in the relevant places. 

What software was used to do the analysis?

Response: We have used R software. Various packages including nlme, sp, sf, lme4, MASS, ggplot2 are used for analyzing and creating graphs and maps.

Where are the sub-district level variables derived from? the DHS itself? specify source and if you derived them, how this was done?

Response: We have mentioned about the data sources in the Data Description section. The 5% sample of the Census data is the main source of calculating sub-district specific contextual variables. Since full census data is not available for academic purpose and also dealing such a big data is not possible at individual research level, only a 5% sample of the census data is permitted from the Bangladesh Bureau of Statistics (BBS) for academic research. This 5% sample of the Census data is also available now online from https://international.ipums.org/international/ upon request.

In appendix table 1, instead of pliterate and other variable names from the computer software, spell these out (e.g. % literate), same with the sub-district level variable names.

Response: As per reviewer’s suggestion we have replaced ”p” by “%” in the relevant percentage type variables. In both S1 Table and S2 Table, we have changed them. Since in R software, a variable name cannot be named with % as a first letter of the variable, we used p for percentage in analysis. 

I don't think the figure 1 is necessary.

Response: As model diagnostics, we showed the figure 1 in the main text. As a standard of model diagnostics, it is recommended to show these diagnostics. However, we are fine to place the Figure 1 as a supplementary Figure. Therefore, we renamed the Figure 1 as Supplementary Figure 1 (S1 Fig) and submitted as supplementary materials.

I think the figures in figure 3 on the right would be better presented as points, vs the line, also I’m not sure from the figure what the solid black line represents.

Response: As per the suggestion, we have presented the right panel plots using dots instead of lines. The solid black line in the left panel plots refers the regression line of the direct estimates on the ELL estimates. This solid line shows how the ELL estimates can regress the direct estimates. Such kind of bias diagnostic plot also indicates how the direct estimates are dispersed due to small sample size in the survey data. 

Figure 4, remove the blue lines between districts, use a thin black line instead. Also the islands on the ocean side of the country are basically illegible because there are so many, would simplified shapefile be a better choice for this presentation?

Response: We have used the updated shape file of Bangladesh available online. The simplified shape-file may be possible for district level but not for sub-district level. For consistency between the maps of district and sub-district levels, we wish to keep the shape-files from same source. Now the black lines reduced the problem of illegibility at the south part of the country.

Have the district and sub-district maps have the same scales so the colors are comparable between the two levels.

Response: The range of prevalence varies by district and sub-district level. The current ranges are based on the minimum and maximum prevalence at their respective levels. As per reviewer’s suggestion, we have made them in the scale of sub-district. The relevant changes are made in the main text accordingly.

I would recommend a formally defined Discussion and Conclusion section of the manuscript

Response: We have divided the Conclusion Section into Discussion and Conclusions sections. The comments of the second reviewer are also embedded in the Discussion section. A summary of the findings are added as the Conclusion section.

“Disaggregated level prevalence of ARI, diarrhoea, and ARI/diarrhoea within a period of two-week preceding the survey among under-5 children in Bangladesh are estimated in this study through implementation of an SAE method for binary type outcome variable. The World Bank ELL method has been adapted for binary response variable by developing a random effects logistic model for each of the indicators. The estimated prevalence of each indicator significantly varied at the considered district and sub-district levels; even the proportions are found double the national level in some districts and sub-districts. Spatial distributions of the indicators indicate children of southern and north-eastern regions are more susceptible to be experienced with the occurrence of diarrhoea and ARI related diseases. The most disaggregated level maps suggest that vulnerable sub-districts spread over both highly and less vulnerable aggregated administrative units (say division and district), particularly higher prevalence of ARI are observed in all regions except those sub-districts very close to the capital city Dhaka. Overall, disaggregated level district and sub-district level prevalence of ARI and diarrhoea might help the policy makers to identify the susceptible hotspots, which in turn help the aid industries in effective interventions at the highly vulnerable disaggregated spots.”

Grammatical notes

p5. change area level method to area level methods

p5. change due to zero observation to due to zero observations

p5. change however not possible to estimate to however it is not possible to estimate

p7. change source of drinking water, sanitation to source of drinking water, and household sanitation

p8, multilevel is misspelled as multilevel

p8. problematic is misspelled as problemtic

p8 change discussed in model development section to discussed in the model development section

Response: Many thanks for identifying the flaws in writing. We have read the revised paper multiple times and checked grammar and spells.

Reviewer #2: 

This is an interesting paper about the application of a small area estimation (SEA) method to child morbidity in Bangladesh. The paper is useful as it extends a technique used by the World Bank for continuous variable (consumption per capita and nutrition Z-score) to dichotomous variables on ARI and diarrhea. The authors apply the method to 2011 data from the BDHS and Bangladesh census.

A few comments:

- It will be good to present more information on applications of SAE to other countries, even if using continuous variable. Particular emphasis could be discussing any validation studies or comparisons of the small area estimates obtained with SAE with estimates obtained from other sources for the small areas or with estimates that came from a census (or sample from a census).

Response: We have incorporated some information and references regarding the applications of SAE methods to public health issues in Bangladesh and other countries. Also we added references of SAE works. As for example:

“The World Bank has been utilising a unit-level SAE method known as ELL after the authors Elbers, Lanjouw, and Lanjouw [19] for poverty and nutrition mapping in many developing countries including Bangladesh [20, 21].”

“..the basic idea can be implemented after developing a generalized linear mixed model (GLMM) more specifically a random effect logistic model for the dichotomous response variable [ 22, 23, 24, 25]. The SAE methods based on GLMM model has been applied to estimate district level institutional births [23] and unmet need for contraception [24] in Ghana, and disaggregated (district- and sub-district) level diarrhoea prevalence in Nepal [25].”

- The authors make a good argumentation on the fitness of the model, however, the figures were barely readable. For a method based largely on figures, it was not good.

Response: In this study, we have applied an SAE method to children morbidity data of Bangladesh and showed how the used ELL method works properly in terms of consistency compared to the design-based direct estimates. 

We focused more on application rather than any methodological development and so simulation study has not been conducted in this study for validation of the ELL method. However, we have included a paragraph regarding possible validation study of the ELL method for the binary response variable. In addition, we referred an empirical validation study of this ELL estimator based on Bangladesh child malnutrition study, where ELL approaches for continuous and dichotomous response variables are compared. 

In the Results section, we showed various model diagnostics of the developed model, since the validation of the model is important due to the prediction of the outcome for census children is based on the developed model. Since the main goal of the research is to analyze the spatial distribution of child morbidity in Bangladesh, we followed the approach of an empirical study where model diagnostics are considered as the validity of the predictions. 

As performance of the ELL method, the direct division level estimates (which are design unbiased and consistent) are compared with those estimates produced from the used ELL method. 

In the discussion section, we have mentioned the problems and the possible future studies regarding validation of the ELL method. 

- In page 15, the authors discuss Table 3. The text says “Table 3 indicates that the mean …” but the estimates presented are from the “Median” column in Table 3.

Response: Thanks for the corrections. We have changed the numbers.

- This application had the benefit of having the DHS and the census sample from the year. It will be good for the authors to elaborate on the application of SAE in other situations of data availability that are more prevalent in developing countries, such as, a number of years of separation between the DHS and a census (and its sample), or having a limited number of common variables in the two data sources.

Response: We have discussed these issues relevant to our study in the Discussion section as a separate paragraph (also some issues raised in first questions are covered). 

“Though testing the validity of a statistical approach is rare due to lack of appropriately detailed data, the ELL method for continuous response variable has been validated by estimating a set of predicted welfare estimates to their true values in the state of Minas Gerais, Brazil [34]. The study findings show that the ELL approach is able to produce estimates of welfare which were quite close to their true values. Such kind of validation study for the binary response variable based ELL method used in this study can be conducted in future using only the survey data where any health indicator variable (say, diarrhoea) is available and the survey data structure permits to have sufficient number of observation at lower administrative units. Das et al. [35] implemented an empirical validation study for the binary response variable based ELL estimator by employing the ELL approach to both height-for-age (HAZ) z-score as continuous response variable and indicator variables (stunted: HAZ < -2.00 and severely stunted: < -3.00) by utilizing linear mixed and GLMM models respectively for prediction purpose. Their study findings show that the ELL-type estimator for binary response variable provides reasonably consistent estimates as the continuous response variable based standard ELL method. However, finding a good GLMM model for the indicator like diarrhoea and ARI is tough due to lack of proper explanatory variables in the census data. This is also a major problem for the area-level model experienced in recent SAE study on diarrhoea prevalence in Bangladesh [27]. As for example, statistics on hand washing during diarrhoeal episodes in children and adults cannot be extracted from any administrative source. In addition, time gap between survey and census year for implementing an SAE study could be an important issue if there are not enough time-invariant variables available in the census data [36].”

# Peer review: Decision Letter 1

**DOI:** 10.1371/journal.pone.0220164.r003

**Authors:** Samson Gebremedhin

27 Apr 2020

Disaggregated Level Child Morbidity in Bangladesh: An Application of Small Area Estimation Method

PONE-D-19-18946R1

Dear Dr. Kawsar,

We are pleased to inform you that your manuscript has been judged scientifically suitable for publication and will be formally accepted for publication once it complies with all outstanding technical requirements.

Within one week, you will receive an e-mail containing information on the amendments required prior to publication. When all required modifications have been addressed, you will receive a formal acceptance letter and your manuscript will proceed to our production department and be scheduled for publication.

Shortly after the formal acceptance letter is sent, an invoice for payment will follow. To ensure an efficient production and billing process, please log into Editorial Manager at https://www.editorialmanager.com/pone/, click the "Update My Information" link at the top of the page, and update your user information. If you have any billing related questions, please contact our Author Billing department directly at authorbilling@plos.org.

If your institution or institutions have a press office, please notify them about your upcoming paper to enable them to help maximize its impact. If they will be preparing press materials for this manuscript, you must inform our press team as soon as possible and no later than 48 hours after receiving the formal acceptance. Your manuscript will remain under strict press embargo until 2 pm Eastern Time on the date of publication. For more information, please contact onepress@plos.org.

With kind regards,

Samson Gebremedhin, PhD

Academic Editor

PLOS ONE

Additional Editor Comments (optional):

Reviewers' comments:

Reviewer's Responses to Questions

**Comments to the Author**

1. If the authors have adequately addressed your comments raised in a previous round of review and you feel that this manuscript is now acceptable for publication, you may indicate that here to bypass the “Comments to the Author” section, enter your conflict of interest statement in the “Confidential to Editor” section, and submit your "Accept" recommendation.

Reviewer #2: All comments have been addressed

**********

2. Is the manuscript technically sound, and do the data support the conclusions?

The manuscript must describe a technically sound piece of scientific research with data that supports the conclusions. Experiments must have been conducted rigorously, with appropriate controls, replication, and sample sizes. The conclusions must be drawn appropriately based on the data presented. 

Reviewer #2: Yes

**********

3. Has the statistical analysis been performed appropriately and rigorously? 

Reviewer #2: Yes

**********

4. Have the authors made all data underlying the findings in their manuscript fully available?

The PLOS Data policy requires authors to make all data underlying the findings described in their manuscript fully available without restriction, with rare exception (please refer to the Data Availability Statement in the manuscript PDF file). The data should be provided as part of the manuscript or its supporting information, or deposited to a public repository. For example, in addition to summary statistics, the data points behind means, medians and variance measures should be available. If there are restrictions on publicly sharing data—e.g. participant privacy or use of data from a third party—those must be specified.

Reviewer #2: Yes

**********

5. Is the manuscript presented in an intelligible fashion and written in standard English?

PLOS ONE does not copyedit accepted manuscripts, so the language in submitted articles must be clear, correct, and unambiguous. Any typographical or grammatical errors should be corrected at revision, so please note any specific errors here.

Reviewer #2: Yes

**********

6. Review Comments to the Author

Please use the space provided to explain your answers to the questions above. You may also include additional comments for the author, including concerns about dual publication, research ethics, or publication ethics. (Please upload your review as an attachment if it exceeds 20,000 characters)

Reviewer #2: Review Comments to the Author:

NO ADDITIONAL COMMENTS.

I feel that the authors have addressed my concerns and the manuscript acceptable for publication.

**********

7. PLOS authors have the option to publish the peer review history of their article (what does this mean?). If published, this will include your full peer review and any attached files.

If you choose “no”, your identity will remain anonymous but your review may still be made public.

**Do you want your identity to be public for this peer review?** For information about this choice, including consent withdrawal, please see our Privacy Policy.

Reviewer #2: Yes: GUSTAVO ANGELES

# Editor report: Acceptance letter

**DOI:** 10.1371/journal.pone.0220164.r004

**Authors:** Samson Gebremedhin

6 May 2020

PONE-D-19-18946R1 

Disaggregated Level Child Morbidity in Bangladesh: An Application of Small Area Estimation Method 

Dear Dr. Kawsar:

I am pleased to inform you that your manuscript has been deemed suitable for publication in PLOS ONE. Congratulations! Your manuscript is now with our production department. 

If your institution or institutions have a press office, please notify them about your upcoming paper at this point, to enable them to help maximize its impact. If they will be preparing press materials for this manuscript, please inform our press team within the next 48 hours. Your manuscript will remain under strict press embargo until 2 pm Eastern Time on the date of publication. For more information please contact onepress@plos.org.

For any other questions or concerns, please email plosone@plos.org. 

Thank you for submitting your work to PLOS ONE.

With kind regards,

PLOS ONE Editorial Office Staff

on behalf of

Dr. Samson Gebremedhin 

Academic Editor

PLOS ONE
